# No Dopamine Cell Loss or Changes in Cytoskeleton Function in Transgenic Mice Expressing Physiological Levels of Wild Type or G2019S Mutant LRRK2 and in Human Fibroblasts

**DOI:** 10.1371/journal.pone.0118947

**Published:** 2015-04-01

**Authors:** Marta Garcia-Miralles, Janaky Coomaraswamy, Karina Häbig, Martin C. Herzig, Natalja Funk, Frank Gillardon, Martina Maisel, Mathias Jucker, Thomas Gasser, Dagmar Galter, Saskia Biskup

**Affiliations:** 1 Department of Neurodegeneration, Hertie-Institute for Clinical Brain Research and DZNE, German Center for Neurodegenerative Diseases, 72076 Tuebingen, Germany; 2 Department of Cellular Neurology, Hertie-Institute for Clinical Brain Research and DZNE, German Center for Neurodegenerative Diseases, 72076 Tuebingen, Germany; 3 Department of Medical Genetics and Applied Genomics, Institute of Human Genetics, University of Tuebingen, 72076 Tuebingen, Germany; 4 Boehringer Ingelheim Pharma GmbH & Co. KG, CNS Research, 88397 Biberach an der Riss, Germany; 5 Department of Neuroscience, Karolinska Institutet, 17177 Stockholm, Sweden; UCL Institute of Neurology, UNITED KINGDOM

## Abstract

Mutations within the *LRRK2* gene have been identified in Parkinson’s disease (PD) patients and have been implicated in the dysfunction of several cellular pathways. Here, we explore how pathogenic mutations and the inhibition of LRRK2 kinase activity affect cytoskeleton dynamics in mouse and human cell systems. We generated and characterized a novel transgenic mouse model expressing physiological levels of human wild type and G2019S-mutant LRRK2. No neuronal loss or neurodegeneration was detected in midbrain dopamine neurons at the age of 12 months. Postnatal hippocampal neurons derived from transgenic mice showed no alterations in the seven parameters examined concerning neurite outgrowth sampled automatically on several hundred neurons using high content imaging. Treatment with the kinase inhibitor LRRK2-IN-1 resulted in no significant changes in the neurite outgrowth. In human fibroblasts we analyzed whether pathogenic LRRK2 mutations change cytoskeleton functions such as cell adhesion. To this end we compared the adhesion characteristics of human skin fibroblasts derived from six PD patients carrying one of three different pathogenic LRRK2 mutations and from four age-matched control individuals. The mutant LRRK2 variants as well as the inhibition of LRRK2 kinase activity did not reveal any significant cell adhesion differences in cultured fibroblasts. In summary, our results in both human and mouse cell systems suggest that neither the expression of wild type or mutant LRRK2, nor the inhibition of LRRK2 kinase activity affect neurite complexity and cellular adhesion.

## Introduction

Mutations in Leucine-Rich Repeat Kinase 2 (LRRK2) are strongly associated with sporadic and autosomal-dominant late-onset Parkinson’s disease (PD) [1,2]. Several pathogenic mutations have been identified [3,4]. The most common mutation is G2019S, accounting for up to 7% of familial PD cases and 1–2% of sporadic late-onset cases depending on the population [1,3–5]. The adjacent codon in the LRRK2 sequence harbors a rare pathogenic mutation detected in familial PD cases, I2020T. These two amino acids, glycine and isoleucine, are part of the serine/threonine kinase domain of LRRK2, whereas other pathogenic mutations have been identified in the second enzymatic domain, the GTPase domain (R1441C, R1441G, R1441H, N1437S). Like other proteins in the ROCO family, LRRK2 comprises a conserved Ras-of-complex (ROC) GTPase domain and a C-terminal of Roc (COR) domain [6], and several protein interaction domains such as ankyrin (ANK), leucine-rich repeat (LRR), and a WD40 which might act as a scaffold for assembly of different protein complexes resulting in the activation of a wide variety of signaling cascades [7]. Interestingly, of the over 40 mutations reported within LRRK2, seven mutations are considered pathogenic, and most of them are located in the kinase and GTPase domains [8].

Major efforts have been undertaken to understand the physiological role of LRRK2 and pathogenic mechanisms leading to PD. Several cellular pathways have been described from *in vitro* and *in vivo* studies to be regulated by LRRK2 [9–12], suggesting a multifunctional role of the protein at a cellular level. The most studied function is the role of LRRK2 at the cytoskeleton, particularly, its involvement in the regulation of neurite outgrowth. MacLeod et al. showed for the first time reduced neurite outgrowth and complexity in rat cortical neurons transfected with G2019S mutant LRRK2 (GS-LRRK2) compared to neurons transfected with wild type LRRK2. This finding was replicated in other cellular and animal models [13–18]. Subsequently, accumulation of F-actin and phosphorylated Ezrin/Radixin/Moesin (ERM) proteins were observed in filopodia of developing neurons expressing the GS-LRRK2 mutant protein [19,20]. It has also been shown that the interaction of LRRK2 with small Rho GTPases is important for the regulation of the actin cytoskeleton, since over expression of Rac1 can rescue the GS-LRRK2 mediated neurite shortening [21,22]. In a different approach the analysis of the LRRK2 interactome has identified several proteins related to the actin cytoskeleton [23].

Taken together, there is accumulating evidence that LRRK2 interacts with cytoskeletal proteins and is involved in the regulation of actin cytoskeleton dynamics and that mutations in the kinase domain interfere with these processes. We asked the question whether inhibition of the kinase activity in wild type LRRK2 or of three different pathogenic LRRK2 mutants interferes with the normal cytoskeleton function and regulation in two different cellular systems: primary hippocampal neurons from transgenic mice and human skin fibroblasts.

To this end, we generated and characterized novel LRRK2 transgenic mouse lines expressing wild type LRRK2 or GS-LRRK2 at physiological levels only in neurons. Primary hippocampal cultures from transgenic mice were used to study neurite outgrowth and branching complexity with or without inhibition of LRRK2 kinase activity. We extended our study to include two additional LRRK2 mutations and used human primary skin fibroblasts obtained from healthy subjects and LRRK2 PD patients. In both cell culture systems we used LRRK2-IN-1, a kinase inhibitor which has been shown to inhibit LRRK2’s kinase function [24].

## Material and Methods

### Ethics statement

Skin biopsies were obtained with written informed consent from all subjects and the local medical ethics committee approved the study (Prof. Dr. med. D. Luft, Ethik-Kommission Medizinische Fakultät, Tuebingen, Germany).

The Regierunspräsidium, Tuebingen, Germany, approved to generate, breed, and sacrifice LRRK2 transgenic mice.

### Generation of LRRK2 transgenic mice

To generate LRRK2 transgenic mice, the human LRRK2 cDNA provided by M. Farrer (Mayo Clinic, Florida) was introduced into the pTSC21 vector with the Thy1.2 promoter (size 9.2 kb) provided by M. Staufenbiel (Novartis, Basel, Switzerland). The G2019S mutation was introduced using the Ultra High-Fidelity DNA Polymerase (Stratagene) and the constructs were sequenced. Plasmid purification and pronuclear injections into C57BL/6 pronuclei were performed by Polygene (Switzerland) and four founder animals per construct were bred to produce stable lines for further analysis.

### Characterization of transgene expression in mice

#### Semi-quantitative RT-PCR

Total RNA was extracted from hemi-brains of LRRK2 transgenic mice at different developmental stages using RNAeasy Lipid Tissue Mini Kit (Qiagen) and treated with RNase-free DNase Set (Qiagen) following the manufacturer’s recommendations. The time points analyzed were embryonic day 14 (E14) and postnatal stages P2, P7, P10, P15 and P21. Semi-quantitative RT-PCR was performed in LightCycler (Roche) with QuantiTect SYBR Green RT-PCR (Qiagen). Briefly, 1μl of total RNA was reverse transcribed and amplified using QuantiTect SYBR Green RT-PCR Master mix (Qiagen) and the following primers: LRRK2-F 5’ TCC CTG CCA TAC GAG ATT ACC 3’; LRRK2-R 5’ GCA CAT TTT TAC GCT CCG ATA 3’; Lrrk2-F 5’ CCA AGC AGA GCA AGC AAA GT 3’; Lrrk2-R 5’ GGC GTA CTG ACA TCG CCT AT 3’ and Hmbs-F 5’ TCG GGG AAA CCT CAA CAC C 3’; Hmbs-R 5’ CCT GGC CCA CAG CAT ACA T 3’. Results are expressed normalized to the housekeeping gene Hmbs.

#### Protein quantification

Freshly prepared lysis buffer (1% Triton X-100, 1X complete protease inhibitor Cocktail [Roche], and 1X Phosphatase inhibitor cocktail [Roche] in PBS) were used to homogenize whole brains or dissected brain regions (cortex [CTX], hippocampus [HC], striatum [STR], brain stem [BS] and midbrain [MB]). The protein lysates were kept on ice for 1h with occasional shaking, centrifuged for 15 min at 14.000 rpm and 4°C, and protein concentration was measured using the BCA protein Assay Kit (Pierce Thermo Scientific) following the manufacturer’s instructions. Samples were loaded on 5, 6, or 7% acrylamide SDS-PAGE or NuPAGE 3–8% Tris-Acetate precast gels (Invitrogen). After protein transfer onto polyvinylidenfluorid (PVDF) membranes (Millipore) overnight at 4°C, membranes were blocked for 1h at RT in 5% milk powder in Tris-buffered saline and 0.1% Tween-20 and incubated overnight at 4°C with primary antibody anti-MID LRRK2, anti-LRRK2 Novus-267 (Novus Biologicals), anti-LRRK2 MJFF2 (Clone c41–2) and MJFF5 (Clone c68–7) (Epitomics), anti-ß-tubulin III or anti-Vinculin (Sigma-Aldrich). Horseradish peroxidase-conjugated secondary antibodies anti-mouse (Jackson Immunoresearch) or anti-rabbit (Dako) and chemiluminiscence HRP substrate (Immobilion Western HRP substrate) were used for detection.

#### In situ hybridization

In situ hybridization was performed as described earlier (Westerlund et al. 2008). Fresh-frozen brains from 11-month-old mice were cryosectioned (14 μm), thawed onto glass slides (SuperFrost; VWR, Stockholm, Sweden) and kept at -20°C until use. Slides were air-dried and hybridized overnight with ^33^P end-labeled oligonucleotide probes complementary to human LRRK2 (Exon 30–31, n = 4315–4363; Exon 35–36, n = 5146–5196; NM_198578.3) and mouse α-synuclein (n = 564–611; NM_001042451.1), which were diluted in hybridization cocktail. Following rinsing and dehydration steps, slides were exposed to radiographic films for analysis.

### Immunohistochemistry and stereological analysis of dopamine cells in midbrain

12- to 13-month-old transgenic and littermate non-transgenic (non-tg) mice were anesthetized with Ketamin (120 mg/Kg) and Sedoxylen (10 mg/Kg) before intra-cardial perfusion with cold PBS and 4% paraformaldehyde (PFA). Brains were post-fixed with 4% PFA overnight at 4°C, cryopreserved in 25% sucrose in PBS at 4°C and stored at -80°C until use. Consecutive coronal cryo-sections (20 μm) were incubated with tyrosine hydroxylase (TH) antibody (Rabbit Polyclonal, 1:1000; Pel-Freez Biologicals). Biotinylated goat anti-rabbit antibody (1:200), VECTASTAIN Elite ABC kit and DAB substrate kit for peroxidases (all from Vector Labs) were used according to the manufacturer’s recommendations to visualize TH positive neurons. Sections were counterstained with cresyl violet, dehydrated and cover slipped with Pertex mounting medium (Medite Gmbh).

Unbiased stereological method and a genotype-blinded system was used to count TH+ and Nissl+ neurons in the SNpc from both hemispheres on every sixth section. For each brain 8 sections were analyzed using the Stereo Investigator Software (MBF Bioscience, Williston, VT, USA) with optical fractionator probe connected to an Axioplan2 inverted microscope (Carl Zeiss) and Axiocam MRm Camera (Carl Zeiss). We used a 40 x 40 μm counting frame size, a 100 x 100 μm grid size, a 16 μm dissector height and 2 μm guard. Statistical analysis was performed with GraphPad Prism V6 (one-way ANOVA with Tukey’s *post hoc* test).

### Primary hippocampal cultures and neurite outgrowth analysis

Hippocampus from P0 transgenic and non-tg mice was dissected and dissociated with 0.25% Trypsin/EDTA (Gibco) for 14 min at 37°C. Cells were seeded on 96-well microplate (BD Falcon) pre-coated with 0.5 mg/ml poly-DL-ornithine hydrobromide (Sigma-Aldrich) in cell culture media consisting of Neurobasal-A (Gibco), GlutaMAX-I supplement (Gibco), B27 supplement (Gibco), ßFGF (5 ng/ml, Sigma-Aldrich) and vehicle control (DMSO) (Sigma-Aldrich) or LRRK2-IN-1 diluted in DMSO (0.1μM). Cell culture media was changed every 2 days until neurons were fixed with 3.7% PFA. At day in vitro (DIV) 3, 7 and 14 neuronal cultures were immunostained with mouse anti-ß-Tubulin, Class III antibody conjugated to Alexa Fluor 488 (1:50; BD Pharmingen) and the nuclear marker Hoechst 33342 (1:2000), both diluted in PBS. Fluorescent images were captured using the BD Pathway 855 High-Content Bioimager (BD Bioscience) at 20X magnification and a montage of 25 adjoining images (5x5) per well was obtained. Following image acquisition and using the BD AttoVision V1.6 Software (BD Bioscience) images were processed and analyzed for neurite outgrowth. Several parameters (see Table 1) were measured and then statistically analyzed with GraphPad Prism V6 (two-way ANOVA with Tukey’s *post hoc* test).

**Table 1:** Neurite outgrowth parameters analyzed with the BD AttoVision^TM^ V1.6 Software from BD Bioscience.

Neurite Outgrowth Parameters	Description
Neurite Maximum Length	Maximum length (pixels) of the longest neurite segment for each cell body.
Neurite Total Length	Total length (pixels) of all segments for each cell body.
Neurite Average Length	Average length (pixels) of all segments for each cell body. (Total length/segment count)
Neurite Tree Count	Number of neurite trees from cell body.
Neurite Segment Count	Total number of segments between node points per cell body. Node points can be from secondary, tertiary, etc., branching or intersection with segments from other cell bodies.
Neurite Branches Count	Total number of primary, secondary, tertiary, etc., branches per cell body.
Neurite Node Point	Total number of points where secondary, tertiary, etc., branches exist per cell body. (Number of segments—number of branches)

### Primary human skin fibroblast culture

Human skin biopsies were obtained after written informed consent from four healthy subjects and from six PD patients carrying one of four different pathogenic LRRK2 mutations (Table 2) [22]. The local medical ethics committee approved the study. Primary skin fibroblasts were isolated and grown in RPMI-1640 cell culture media (Biochrom AG) with 15% fetal bovine serum (PAA), 1% penicillin/streptomycin (Biochrom AG) and 1.1% sodium pyruvate (Sigma-Aldrich) at 37°C in 5% CO_2_. Culture media was changed every three-four days and cellular cultures were passaged when cells reached 85–95% confluence. All experiments were performed at passages 9–10.

**Table 2:** List of primary human skin fibroblasts from healthy control subjects and LRRK2-PD patients.

	Identification Number	Sex	Age at biopsy	Age onset	Mutation	Protein Domain
**Healthy Subjects**	ID16423	F	77	-	-	-
	ID16424	M	62	-	-	-
	ID16425	F	80	-	-	-
	ID16392	M	61	-	-	-
**LRRK2-PD Patients**	DNA13287	M	58	55	G2019S	Kinase Domain
	DNA14694	F	62	53	G2019S	Kinase Domain
	DNA12098	F	77	70	G2019S	Kinase Domain
	DNA9236	F	61	39	R1441C	GTPase Domain
	DNA10688	M	42	38	N1437S	GTPase Domain
	DNA10689	M	68	48	N1437S	GTPase Domain

(F, female; M, male)

For each fibroblast line, 2000 cells/well were plated on 96-well microplate (BD Falcon) and incubated at 37°C and 5% CO_2_. After 10, 30, 60, 120 and 180 min of incubation, cells were rinsed twice with PBS to dispose of unattached cells. Attached cells were fixed for 1 min with Accustain (Sigma) and stained with the nuclear marker Hoechst 33342 diluted in PBS (1:5000) for 10 min at RT. For every cell line and experiment, a seeding control was included, in which cells were incubated for 180 min and fixed without rinsing with PBS to maintain the maximum number of attached cells. All time points and cell lines were analyzed in duplicates in each independent experiment.

To analyze the effect of LRRK2 kinase inhibition on cell adhesion capacity, we used LRRK2-IN-1 in DMSO. Fibroblasts were seeded in duplicate in 96-well plates and the cell culture medium was supplemented with vehicle (DMSO), or 0.1 or 1 μM LRRK2-IN-1 in DMSO. After 30 or 120 min cells were rinsed twice with PBS, fixed for 1 min with Accustain and stained for Hoechst 33342 following the same procedure as above.

### Cell counting

Fluorescent images were captured using the BD Pathway 855 High-Content Bioimager (BD Bioscience) at 20X magnification and a montage of 25 not adjoining images (5x5 with 1 mm gap) for each well. Images were first processed with the BD AttoVision V1.6 Software (BD Bioscience), and the intensity of Hoechst 33342 staining was used as parameter to count the number of attached cells. The percentage of attached fibroblasts per well was calculated from total number of seeded cells per well. The graphs represent mean values from 3 or 4 independent experiments and statistics were analyzed with GraphPad Prism V6 (two-way ANOVA with repeated measures or two-way ANOVA with Tukey’s *post hoc* test).

### Protein quantification

Primary skin fibroblasts were homogenized in freshly prepared lysis buffer, rotated in a wheel for 1h at 12 rpm and 4°C, centrifuged for 15 min at 14.000 rpm at 4°C, and protein concentration was determined as described above. Samples were resolved using 5% acrylamide SDS-PAGE, transfer onto polyvinylidenfluorid (PDVF) membranes (Millipore) and incubated overnight at 4°C with primary antibody anti-LRRK2 MJFF2 (Clone c41–2) and anti-ß-tubulin III (Sigma-Aldrich). Horseradish peroxidase-conjugated secondary antibodies anti-mouse (Jackson Immunoresearch) or anti-rabbit (Dako) and chemiluminiscence HRP substrate (Immobilion Western HRP substrate) were used for detection.

## Results

### Generation of Human Wild Type and G2019S Mutant Thy1.2-LRRK2 Transgenic Mice

**Fig 1:**
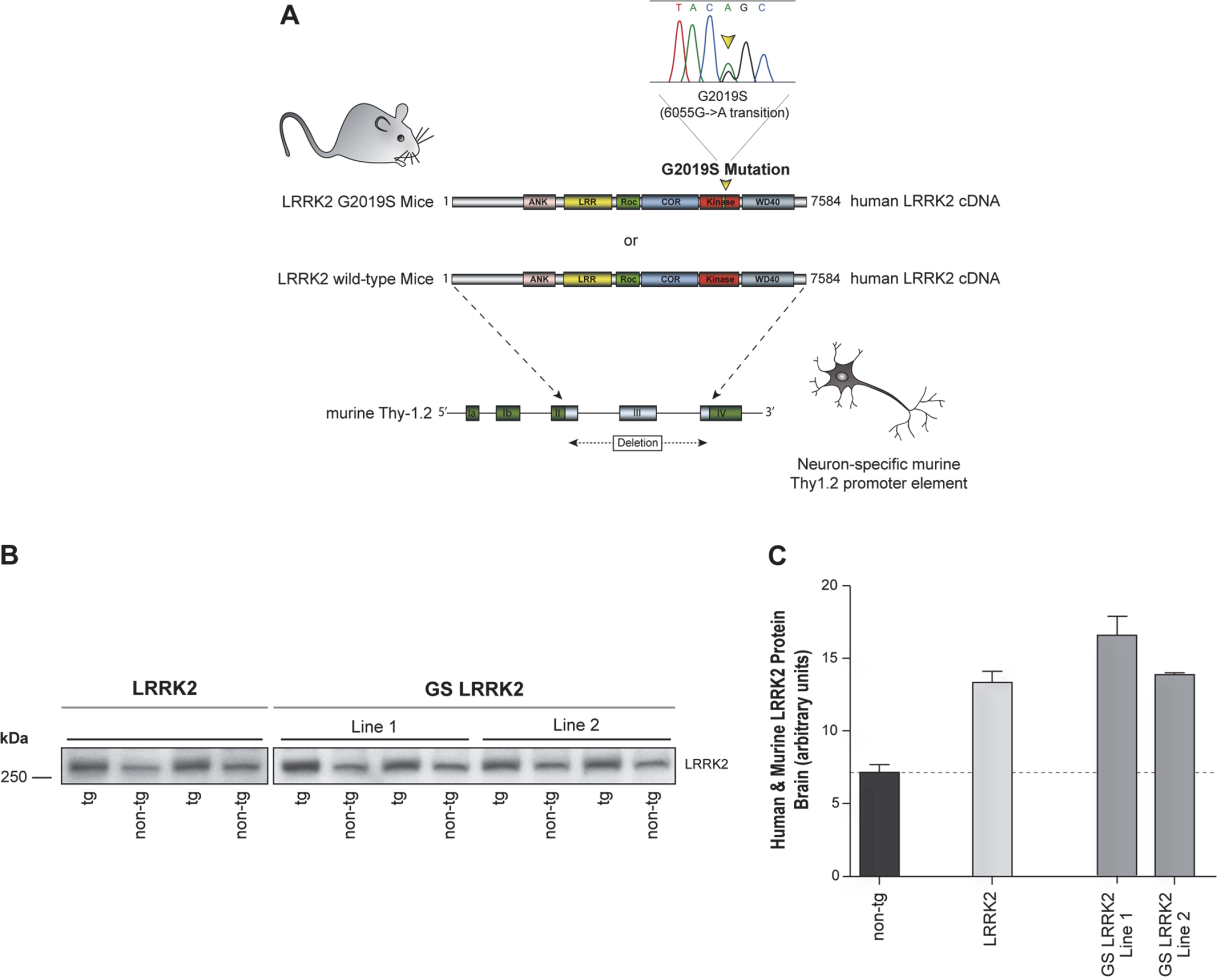
Generation of human wild type and G2019S mutant LRRK2 transgenic mice. **A:** Human full length wild type or G2019S mutant LRRK2 cDNA were cloned into the murine Thy-1.2 promoter element driving neuronal-specific transgene expression. **B:** Western blot analysis of LRRK2 protein expression in brain lysates from LRRK2, GS-LRRK2 line 1, GS-LRRK2 line 2 and non-tg littermates using MID antibody which recognizes human and murine LRRK2 protein. **C:** Densitometry quantification revealed approximately twice the amount of total LRRK2 protein in all transgenic lines compared to endogenous Lrrk2 levels in non-tg controls. Data represent means ± SEM; n = 2 for each transgenic line.

To generate these novel transgenic mouse lines, the cDNA comprising all 51 exons of the human wild type or mutated LRRK2 protein were sub-cloned into the mini-gene version of the murine Thy-1.2 promoter expression cassette [25] to induce neuronal-specific expression of the transgene in mice (Fig. 1A). Final expression constructs were microinjected into C57BL/6 oocytes in order to produce LRRK2 transgenic mice.

Three transgenic lines revealed significant and similar stable transgene expression levels, one LRRK2 line, and two lines expressing the G2019S mutant LRRK2 (GS-LRRK2 line 1 and GS-LRRK2 line 2). Brain lysates from 4-month-old mice of the F2 generation were used to quantify LRRK2 protein levels (Fig. 1B). The MID antibody [26] directed to the middle region of the protein (amino acids 801–1000) detects both the human and the murine LRRK2 protein. Densitometry quantitation of total LRRK2 protein showed a roughly two-fold expression in all three transgenic lines compared to the non-transgenic line, and indicates that the human transgene is expressed at the same level as the murine endogenous Lrrk2 (assuming that the antibody recognizes the two homolog proteins with similar affinity, Fig. 1C). All transgenic mice from the selected stable lines were fertile and viable into adulthood. They did not present any obvious behavioral phenotype or changes in body weight and length compared to non-transgenic control mice up to 18 months of age (data not shown).

### Characterization of transgene expression in the novel Thy1.2-LRRK2 transgenic mouse models

The further characterization of LRRK2 transgene expression in the novel Thy1.2-LRRK2 transgenic mouse lines included three different assessments: analysis of transgene expression during embryonic and postnatal development; analysis of the cellular localization pattern of the transgene in the brain, in particular in those regions affected by PD neuropathology; and analysis of the number of dopamine neurons in the midbrain to detect PD-associated neuropathology.

**Fig 2:**
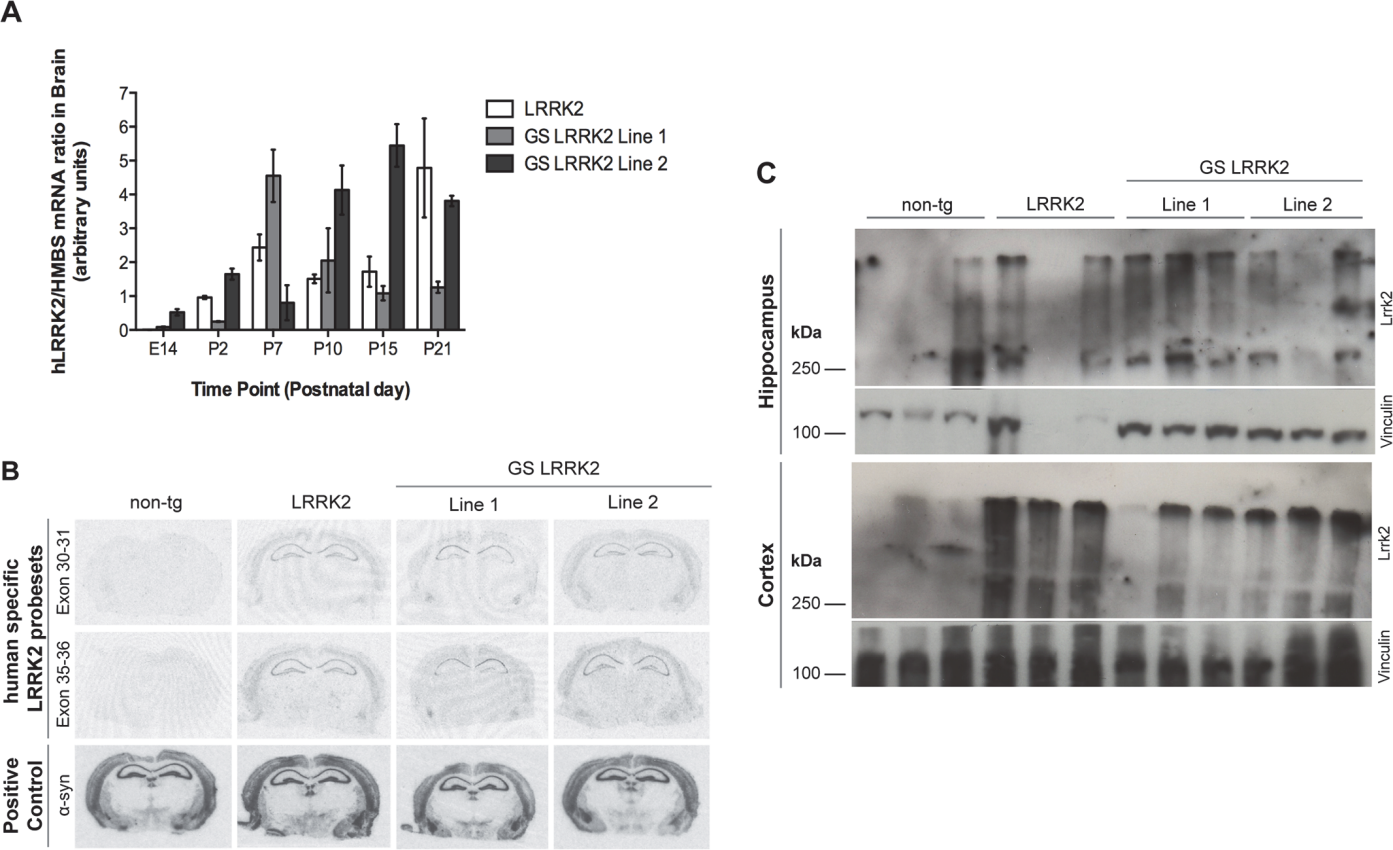
LRRK2 mRNA and protein expression in brain regions in the three transgenic mouse lines. **A:** RT-PCR semi-quantification of LRRK2 expression in whole brains at different embryonic and postnatal stages indicates robust transgene expression at postnatal day 2 (P2) in all three lines. Data represents means ± SEM; n = 3–5 animals per group. **B:** In-situ hybridization of coronal brain sections at the level of posterior hippocampus and midbrain with two human specific LRRK2 probes showed comparable transgene expression levels in hippocampus and cortex of 11-month-old LRRK2 and GS-LRRK2 lines 1 and 2. **C:** Western blot analysis of LRRK2 protein showed robust expression levels of LRRK2 in hippocampus (HC) and cortex (CTX) of 10-month-old animals with the human-specific LRRK2 antibody MJFF5; n = 3 animals per genotype.

The temporal expression profile of LRRK2 transgene in the brain was studied by semi-quantitative RT-PCR (Fig. 2A). At E14, the earliest time point analyzed, the expression of LRRK2 was detectable only in the GS-LRRK2 line 2. In early postnatal days the amount of LRRK2 mRNA is readily detectable in all three mouse lines and increased during the following weeks. Interestingly, in two transgenic lines (LRRK2 and GS-LRRK2 line 2) the expression levels increased steadily from P2 to P21, whereas in the GS-LRRK2 line 1 a high level of transgene is reached already at P7 and at later time points (P10, P15 and P21) the expression levels decline gradually to clearly detectable but low signal.

To establish whether the transgene is expressed in brain regions important for the neuropathology of PD, we studied the cellular localization of LRRK2 in the transgenic mice. We used in situ hybridization with two different human specific oligonucleotide probes able to distinguish between the endogenous Lrrk2 and the human transgene (Fig. 2B). To model the late age at onset of PD pathology we analyzed 11-month-old mice from all three transgenic lines as well as non-transgenic littermates. The level and expression pattern of α-synuclein were similar for all four lines (lower row) and show a distinct signal in cortex, hippocampus, striatum, and substantia nigra. Adjacent sections were hybridized with the LRRK2 probes detecting no signal in non-transgenic mice and comparable expression levels and patterns of the human LRRK2 transgene in the hippocampus and cerebral cortex in all three transgenic lines. Reflecting perhaps the lower transgene levels in 3-week-old mice from the GS-LRRK2 line 1, we detected lower levels of LRRK2 mRNA in this line, in particular in the dentate gyrus and hippocampus. No transgene mRNA expression was detected in striatum and *Substantia nigra pars compacta* (SNpc), consistent with the very low levels of LRRK2 protein in these regions detected by Western blot in 10-month-old transgenic mice (Fig. 2B and S1 Fig.). High levels of LRRK2 protein were found in the hippocampus and cortex in all three transgenic lines with the human-specific MJFF5 LRRK2 antibody (Fig. 2C).

**Fig 3:**
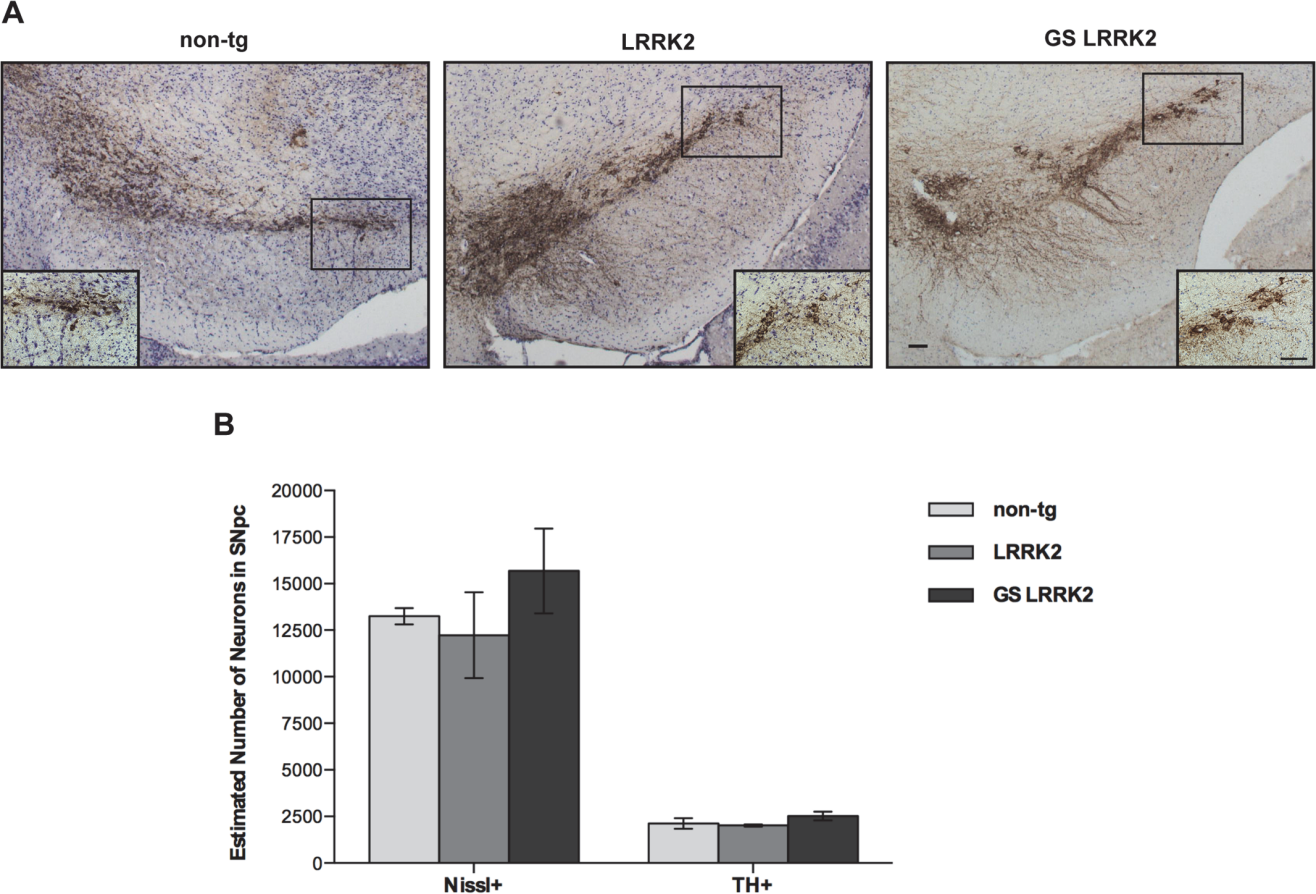
No neuronal loss or neurodegeneration was detected in SNpc in transgenic mice. **A:** Representative coronal section of midbrain sections from 12- to 13-month-old non-tg, LRRK2 and GS-LRRK2 (line 2) transgenic mice immunostained for TH. (Scale bars: 100μm). **B:** Cell counts of TH+ and Nissl+ neurons in SNpc from non-tg, LRRK2 and GS-LRRK2 transgenic mice. Data represent mean ± SEM; transgenic mice n = 2, non-tg mice n = 3. *n*.*s* = not significant, (one-way ANOVA, Tukey’s *post hoc* analysis).

Finally, we determined whether overexpression of human LRRK2 protein causes loss and/or degeneration of DA neurons in SNpc in these transgenic mice. An unbiased stereological method was applied to quantify the number of tyrosine hydroxylase positive (TH+) and Nissl positive (Nissl+) neurons in SNpc. At the age of 12–13 months we observed no obvious differences in TH+ or Nissl+ cells in the transgenic mice compared to non-tg littermates (Fig. 3A). These observations were confirmed by the cell counts indicating no significant difference between the mouse lines (*n*.*s*, one-way ANOVA with Tukey’s *post hoc* analysis, Fig. 3B). In summary, our results suggest that overexpression of LRRK2 is not sufficient to cause loss or neurodegeneration of DA neurons in these particular transgenic mouse lines.

### Modulation of Neurite Outgrowth by LRRK2 mutations and kinase inhibition

Previous studies indicating that LRRK2 is involved in the regulation of neurite outgrowth and branching complexity were performed in transfected cell cultures or animal models expressing high and variable levels of LRRK2 [14–17]. Our goal was to investigate in detail the effect of wild type and mutated LRRK2 expressed at physiological levels, comparable to the endogenous murine LRRK2 levels, in the modulation of neurite outgrowth, and whether LRRK2 kinase activity impacts this process.

**Fig 4:**
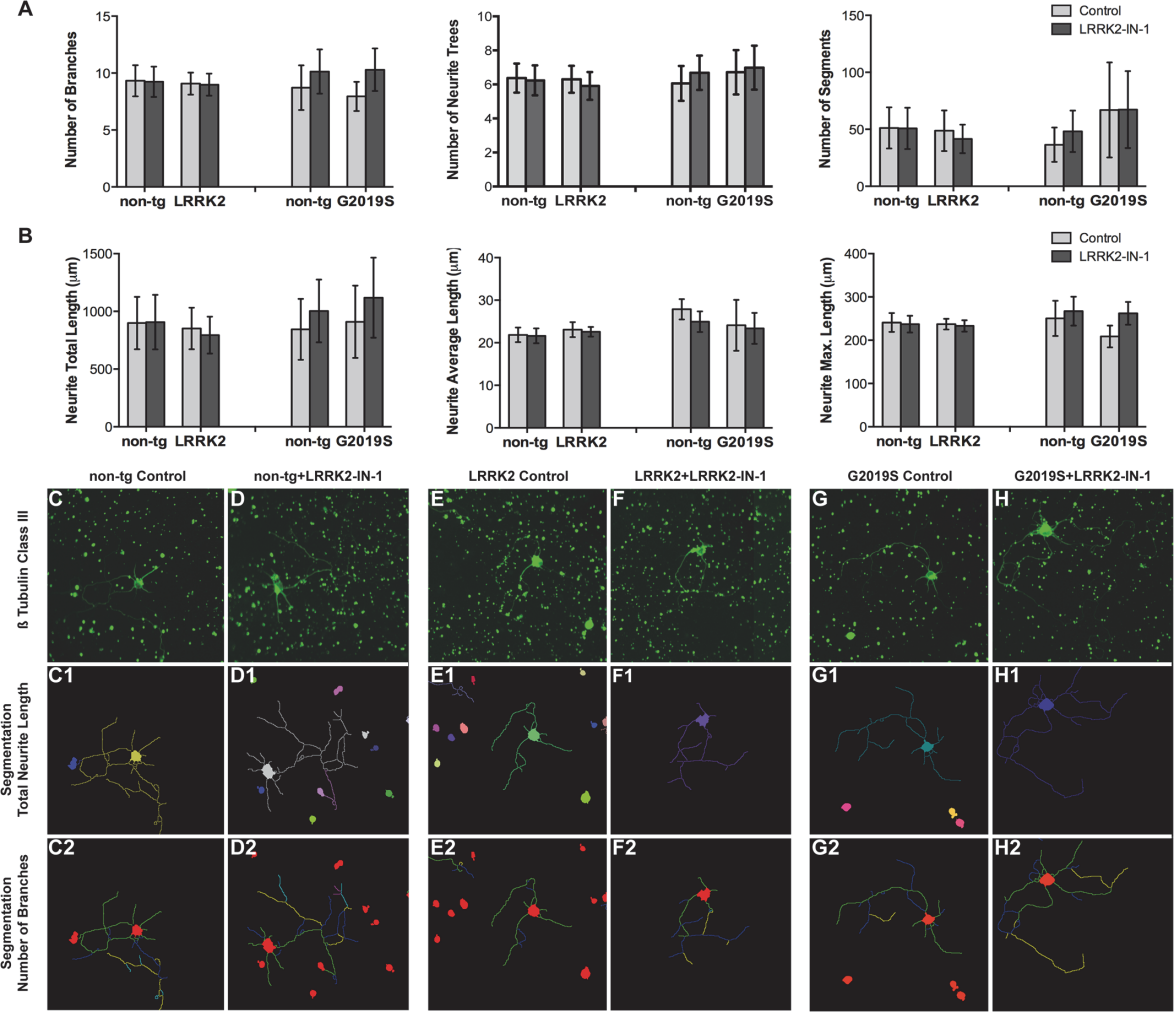
Analysis of neurite outgrowth and branching complexity of primary hippocampal neurons. **A-B:** Neurite parameters of neuronal cultures from LRRK2, GS-LRRK2 (line 2) and their respective non-tg littermate, which were treated with vehicle-control or LRRK2-IN-1 (0.1 M) for seven days (DIV7). Comparison of parameters describing neurite branching (A) included number of branches, number of neurite trees and number of segments. Comparison of neurite length parameters (B) included total neurite length, average neurite length and maximal neurite length. Data represent mean ± SEM and were analyzed with two-way ANOVA. No significant difference was detected. Number of neurons analyzed for cultures obtained from LRRK2 transgenic mice: non-tg = 1339, non-tg + LRRK2-IN-1 = 1609; LRRK2 = 1697, LRRK2 + LRRK2-IN-1 = 1542, n = 4 independent experiments; Number of neurons analyzed for cultures obtained from GS-LRRK2 transgenic mice: non-tg = 1268; non-tg + LRRK2-IN-1 = 1522; GS-LRRK2 = 1526; GS-LRRK2 + LRRK2-IN-1 = 1844, n = 4 independent experiments; **C-H:** Representative pictures of ß-Tubulin III stained neurons on DIV7 derived from wild type, GS- LRRK2 (line 2), their non-transgenic littermates. Pictures were obtained with the BD Pathway 855 high content Bioimager. **C1-H2:** Total neurite length (C1-H1) and number of branches (C2-H2) segmentation corresponding to ß-tubulin III staining images (C-H) obtained from Attovision Software.

To this end, primary hippocampal neurons were prepared from newborn pups from non-transgenic, LRRK2, and GS-LRRK2 mouse lines. Two different non-transgenic cell cultures were included derived from the littermates of the LRRK2 line or the GS-LRRK2 mouse line 2, respectively. One half of the primary cultures were treated with vehicle control (DMSO) whereas the parallel cultures were treated with 0.1 μM of the LRRK2 kinase inhibitor LRRK2-IN-1. This concentration was tested previously in mouse fibroblast and showed inhibition of LRRK2 kinase activity by reducing 22% of LRRK2 phosphorylation at residue S935 (S3 Fig.). For all cell cultures we analyzed seven different neurite parameters on three days in vitro (DIV3, DIV7 and DIV14). The neurite characteristics studied with the BD AttoVision Software are described in Table 1. While all data are available in S2 Fig., we focus on the results on DIV7. Following two-way ANOVA post hoc tests (Table A-C in S1 File), we compared neurite parameters on DIV7 between LRRK2 or GS-LRRK2 expressing neurons and their corresponding non-transgenic littermate neuronal cultures under vehicle treated conditions, however, no significant changes were observed in parameters related to branching complexity (Fig. 4A) nor in parameters related to neurite length (Fig. 4B). We also analyzed the effect of the kinase inhibitor and found no significant changes (Fig. 4A and B). Human LRRK2 protein expression was assessed by Western blot on DIV3 and DIV7 in G2019S LRRK2 primary hippocampal cultures and their non-tg littermate controls (S4 A Fig.). Human LRRK2 mRNA expression was verified in wild type LRRK2 primary hippocampal cultures on DIV7 by RT-PCR (S4 B Fig.).

### Treatment with the LRRK2-IN-1 kinase inhibitor does not alter adhesion properties in LRRK2 primary human skin fibroblasts

**Fig 5:**
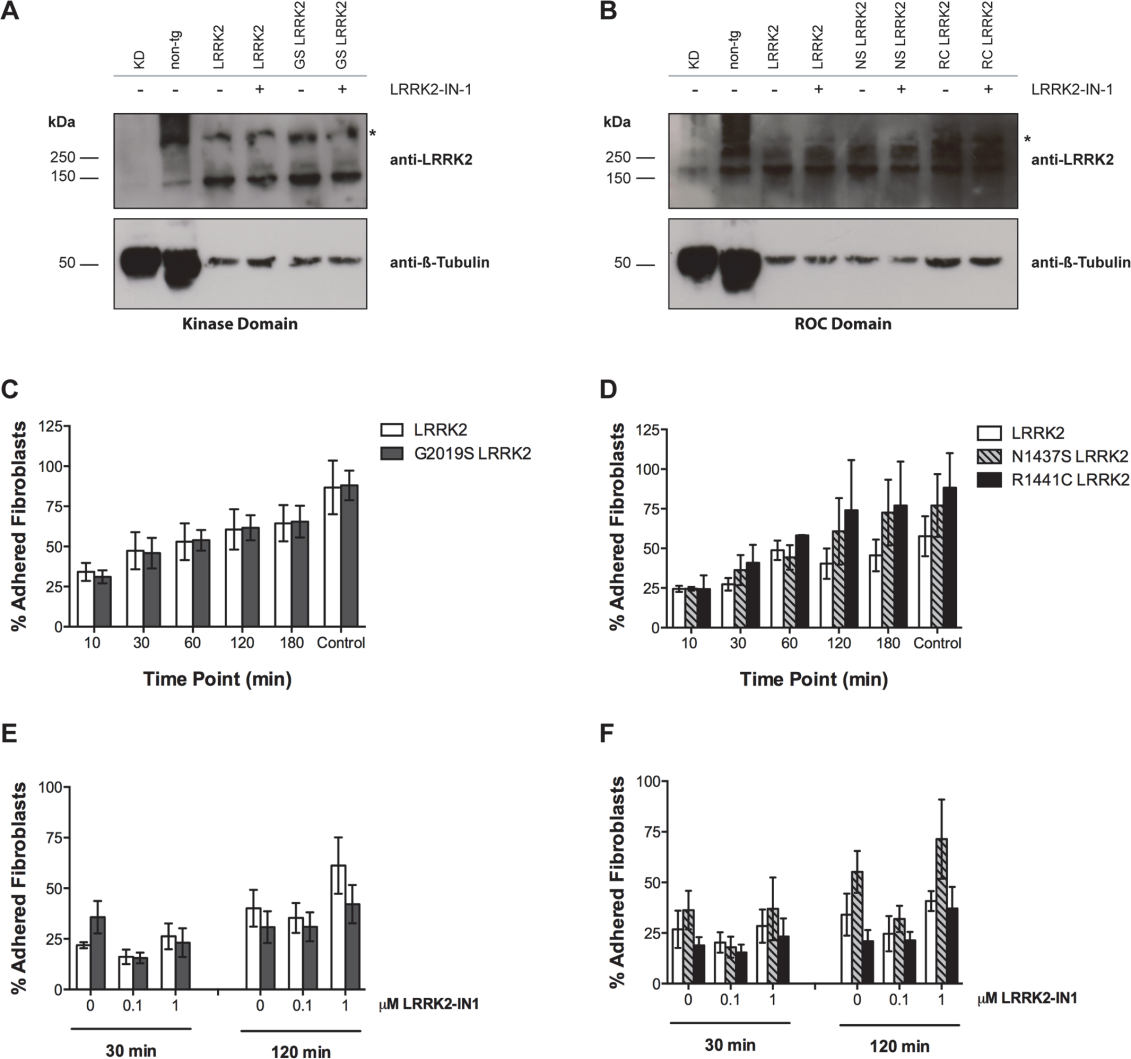
Treatment with the LRRK2-IN-1 kinase inhibitor does not alter adhesion properties in LRRK2 primary human skin fibroblasts. **A-B:** Western blot analysis of LRRK2 expression in primary skin fibroblasts from healthy subjects (LRRK2) and PD-patients with mutations in the kinase (A) and ROC domain (B) of LRRK2 after treatment with vehicle or 0.1μM LRRK2 IN-1. The MJFF#2 antibody we used recognizes both human and mouse LRRK2. Brain lysates from a Lrrk2 knock-down mouse (KD) and cortex lysate from a non-tg mouse served as negative and positive controls, respectively. One representative fibroblast line per mutation group is shown and 30μg protein was loaded on a 7% acrylamide SDS-gel. **C-D:** Percentage of adhered fibroblasts with LRRK2 mutations in the kinase (C) and ROC (D) domain at different time points. No significant differences in adhesion capacity were observed between lines. **E-F**: Percentage of adhered fibroblasts with LRRK2 mutations in the kinase (E) and ROC (F) domain after treatment with vehicle control (0), 0.1μM or 1μM LRRK2-IN-1 for 30 and 120 minutes. No differences were observed in fibroblasts with LRRK2 mutations in the kinase (E) and ROC domain (F). Data represent mean ± SEM; n = 4 independent experiments (C, E) and n = 3 independent experiments (D, F). Healthy-Subjects (LRRK2) = 4 lines; G2019S LRRK2 patients (GS) = 3 lines; N1437S LRRK2 patient (NS) = 2 lines; R1441C LRRK2 patients (RC) = 1 line. (Two-way ANOVA with Repeated Measures).

Based on previous results we were interested if the inhibition of LRRK2 kinase activity affects cytoskeleton function in cells expressing pathological LRRK2 mutations. We used a different cell culture model with physiological levels of LRRK2: fibroblasts prepared from skin biopsies from PD patients carrying one of three different pathogenic LRRK2 mutations, and from healthy controls carrying wild type LRRK2. The effect of LRRK2 mutations and kinase inhibition on cytoskeleton dynamics was studied as a function of cell adhesion at the indicated time point and is expressed as percentage attached cells of all plated cells. Fibroblasts from six PD patients carrying either a mutation in the kinase domain (G2019S) or in the ROC domain (N1437S or R1441C) and four age-matched healthy controls were included in the study (Table 2). We first verified that each of the fibroblast lines expressed LRRK2 in comparable amounts and that the LRRK2-IN-1 treatment did not alter expression levels (Fig. 5A and B). Next, we compared cell adhesion of newly plated cells at 10, 30, 60, 120, and 180 minutes after cell plating and found no significant difference (Table D in S1 File) between fibroblasts from healthy-controls and fibroblasts from carriers of LRRK2 mutations in the kinase domain (Fig. 5C) or in the ROC domain (Fig. 5D).

Finally, we compared the effect of the kinase inhibitor LRRK2-IN-1 on cell adhesion at two time points, 30 and 120 minutes, but observed no significant differences (Table E in S1 File) between healthy-control and LRRK2 mutant fibroblast (Fig. 5E and F). All data regarding individual fibroblast lines is available at supporting information (S5 Fig.).

In summary, our findings show that the three pathogenic LRRK2 mutations do not induce significant changes in cell adhesion compared to wild type LRRK2, and that inhibition of kinase activity does not have an impact on cell adhesion of mutant or wild type LRRK2 carriers.

## Discussion

One of the first reported cellular phenotype of mutated LRRK2 is the regulation of neurite morphology [14]. The Abeliovich team used primary cortical rat cultures transfected with wild type or mutated human LRRK2 to show that G2019S LRRK2 overexpression induced reduction of neurite length and branching, and additionally underline that LRRK2 silencing induced the opposite effect [14]. The roughly 30% reduction of neurite length in G2019S cultures is a very robust phenotype that has been reproduced in several cell systems and has recently been used as read out to demonstrate the interaction between RAB7L1 and LRRK2 [27]. A study from the Farrer laboratory used primary hippocampal cultures derived from BAC transgenic mice to compare cellular effects of LRRK2 mutations confirming the mutation specific drop in neurite branching and length in cultures from G2019S mice [13]. Although the protein load of these BAC transgenic mice was very high, exceeding 10 to 20 times the endogenous mouse LRRK2 levels, cultures from mice expressing wild type LRRK2 had comparable neuritic arborizations on day 8 in culture as cultures from non-transgenic littermates.

The novel mouse model we generated and characterized in this study induces moderate levels of LRRK2 expression that do not exceed a 2-fold overexpression of endogenous LRRK2 levels. The neuronal hippocampal cultures derived from these mice were analyzed with an automated system on day 7 in culture and we detected no significant changes in neurite length or branching parameters. Our results are in line with the findings from hippocampal and dopamine neuronal cultures derived from murine G2019S-LRRK2 knock-in mice, which show no impairment of neurite parameters [13]. Similarly, a recent study using hippocampal neurons derived from BAC transgenic mice encoding LRRK2 and LRRK2-G2019S and expressing approximately six-fold higher LRRK2 brain levels showed only transient reduction of neurite length and branching over the course of three weeks [28]. Interestingly, the authors also demonstrated a clear influence of growth substrate with slower growth on poly-L-Lysine and rapid growth on laminin coated cell culture surfaces. Using time lapse imaging a significant decrease of growth cone motility was detected in neurons derived from LRRK2 transgenic mice although these differences did not translate into changes in neurite length. Taken together, the results demonstrate that the drop in neurite growth and branching is dependent on very high levels of mutant LRRK2 and is independent of neuronal cell type or species.

The G2019S mutation is generally accepted to induce increased kinase activity and accordingly, treatment with unspecific kinase inhibitors, such as staurosporine, have been shown to normalize the drop in neurite arborization induced by high levels of mutated LRRK2 [13]. However, we did not observe any change in neurite outgrowth after treating our primary hippocampal neurons with the LRRK2-IN-1. One possible explanation to this observation can be the recently described off-target effects of LRRK2-IN-1 in several cellular systems. Specifically related to neurite outgrowth it was shown that endogenous wild type and knockout LRRK2 primary neuronal cultures treated with the LRRK2-IN-1 displayed a similar neurite outgrowth phenotype mediated independently of LRRK2 [29].

Three new mouse lines have been characterized in detail in this study, one line expressing wild type LRRK2 and two lines expressing G2019S LRRK2 downstream the neuron specific Thy1.2 promoter. The localization of LRRK2 mRNA and protein expression was analyzed in selected developmental stages and in adult animals and compared to the murine LRRK2 expression in particular in those brain areas affected by PD [30–33]. The careful analysis of dopamine neurons (which show no transgene expression) in our new mouse lines indicated no neuronal loss or neurodegeneration of dopamine neurons at 12 months of age. These results are in line with findings in other transgenic mice expressing wild type or mutated LRRK2 downstream different promoters, indicating that plainly LRRK2 expression does not precipitate neuro-pathological phenotypes characteristic for PD, at least not in the mouse system [17,34–36].

We extended our study on the cellular effects of LRRK2 mutations expressed at physiological levels to analyze primary human skin fibroblasts derived from PD patients and healthy wild type LRRK2 carriers. Primary skin fibroblasts represent a convenient cell model for neurodegenerative diseases, which have previously been used to study effects of PINK and Parkin mutations on mitochondrial function [37]. We used the fibroblast cultures to study cellular adhesion, a process primarily regulated by small GTPases from the Rho family which control actin polymerization and activation of myosin II [38]. LRRK2 has previously been shown to interact with members of the Rho family [21,22] and has a potential role in actin cytoskeleton dynamics. The fibroblast adhesion test has earlier been used in two other neurological disorders, in Alzheimer disease and in Lesch Nyham disease, and in both studies cellular adhesion properties of patient fibroblasts were altered, being decreased and increased respectively [39,40]. In our study we detected no alteration in the adhesion of fibroblasts from Parkinson patients carrying LRRK2 mutations in the kinase domain or in the ROC domain compared to fibroblasts from healthy controls carrying wild type LRRK2.

In conclusion, we show in two different cellular models that both wild type and mutant LRRK2 protein expressed at physiological levels do not compromise the regulation of actin cytoskeleton arrangements and/or dynamics. Given the tremendous variability of reported functions for LRRK2 protein in various cellular and animal overexpression models, the results of our study are particularly relevant as (i) we investigate the function of the protein LRRK2 in a novel in depth characterized animal model, and (ii) we investigate models with endogenous LRRK2 levels or moderate overexpression levels of LRRK2 and hereby reproduce conditions of the wild type and mutated protein in a physiological environment.

## Supporting Information

S1 Fig LRRK2 protein expression in different brain regions in the three transgenic mouse lines. Western blot analysis of LRRK2 protein showed expression levels of LRRK2 in brainstem (BS), midbrain (MB), but not in striatum (STM) of 10-month-old animals with the human-specific LRRK2 antibody Novus 267. n = 3 animals per genotype.
(TIF)

S2 Fig Analysis of all neurite outgrowth and branching complexity parameters studied in primary hippocampal neurons. A-G: Neurite parameters analyzed in neuronal cultures from LRRK2, GS-LRRK2 (line 2), and their respective non-tg littermate neurons which were treated with vehicle-control or LRRK2-IN-1 (0.1 μM) for three (DIV3), seven (DIV7), or fourteen days (DIV14). Data represent mean ± SEM; Two-way ANOVA (* p<0.05; ** p<0.01); Number of neurons analyzed for cultures obtained from LRRK2 transgenic mice: non-tg = 1339, non-tg + LRRK2-IN-1 = 1609; wild type LRRK2 = 1697, wild type LRRK2 + LRRK2-IN-1 = 1542, n = 4 independent experiments; Number of neurons analyzed for cultures obtained from GS-LRRK2 transgenic mice: non-tg = 1268; non-tg + LRRK2-IN-1 = 1522; GS-LRRK2 = 1526; GS-LRRK2 + LRRK2-IN-1 = 1844, n = 4 independent experiments.
(TIF)

S3 Fig LRRK2-IN1 inhibits LRRK2 kinase activity by reducing phosphorylation at residue S935 in mouse fibroblasts. A: Western blot analysis showing reduction of endogenous LRRK2 phosphorylation at residue S935 in mouse fibroblasts after incubation with LRRK2-IN1 inhibitor at different concentrations (0, 0.1, and 0.3 μM) for 20 min. Inhibition of LRRK2 kinase activity can be observed at 0.1 μM of LRRK2-IN1 by reducing LRRK2 phosphorylation at residue S935 (phosphospecific LRRK2 antibody against residue pS935). B: Densitometry quantification revealed approximately 22% reduction of pS935 LRRK2 protein after incubation with 0.1μM of LRRK2-IN1.
(TIF)

S4 Fig LRRK2 protein and mRNA expression in LRRK2 Primary Hippocampal Cultures. A: Western blot analysis of human LRRK2 protein expression in GS-LRRK2 primary hippocampal cultures at DIV1, 3, 5 and 7 derived from GS-LRRK2 transgenic newborn pups. Human LRRK2 protein is only expressed in GS-LRRK2 primary hippocampal neurons but not in their non-tg littermate controls (human-specific anti-LRRK2 antibody MJFF5). B: RT-PCR semi-quantification of human LRRK2 mRNA expression in LRRK2 primary hippocampal cultures at DIV7 derived from LRRK2 transgenic newborn pups and their respective non-tg littermates. Whole brain lysate from non-transgenic and LRRK2 transgenic mice were used as negative and positive control, respectively. Data represents mean ± SEM. Number of newborn pups: non-tg = 5, LRRK2 = 7; Number of control samples: negative = 2, positive = 2; n = 2 independent experiments.
(JPG)

S5 Fig Results of cellular adhesion assays from individual fibroblast lines derived from healthy-control subjects and patients with kinase or GTPase mutations without or with LRRK2-IN-1 kinase inhibitor treatment. A-B: Percentage of adhered fibroblasts with wild type LRRK2 (healthy subject lines) and LRRK2 mutations in the kinase (A) and ROC (B) domain at different time points. C-D: Percentage of adhered fibroblasts with wild type LRRK2 (healthy subject lines) and LRRK2 mutations in the kinase (C) and ROC (D) domain after treatment with vehicle control (0), 0.1μM or 1μM LRRK2-IN-1 for 30 and 120 minutes. Data represent mean ± SEM; n = 4 independent experiments (A, B) and n = 3 independent experiments (C, D). Healthy-Subjects (wild type LRRK2) = 4 lines; G2019S LRRK2 patients (GS) = 3 lines; N1437S LRRK2 patient (NS) = 2 lines; R1441C LRRK2 patients (RC) = 1 line. (Two-way ANOVA with Repeated Measures).
(TIF)

S1 File Supporting tables. Table A, Two-way ANOVA statistical results from primary hippocampal neurons at DIV3. Table B, Two-way ANOVA statistical results from primary hippocampal neurons at DIV7. Table C, Two-way ANOVA statistical results from primary hippocampal neurons at DIV14. Table D, Two-way ANOVA statistical results from cellular adhesion assay timeline. Table E, Two-way ANOVA statistical results from cellular adhesion assay LRRK2-IN-1 treatment.
(DOCX)

S1 Protocol A, LRRK2 protein quantification in primary hippocampal cultures. B, LRRK2 kinase inhibition by LRRK2-IN1 in mouse fibroblasts.
(DOCX)

## References

[1] Gasser T, Hardy J, Mizuno Y (2011) Milestones in PD genetics. Movement disorders: official journal of the Movement Disorder Society 26: 1042–1048. 10.1002/mds.23637 21626549

[2] Paisan-Ruiz C (2009) LRRK2 gene variation and its contribution to Parkinson disease. Hum Mutat 30: 1153–1160. 10.1002/humu.21038 19472409

[3] Bekris LM, Mata IF, Zabetian CP (2010) The genetics of Parkinson disease. Journal of geriatric psychiatry and neurology 23: 228–242. 10.1177/0891988710383572 20938043 PMC3044594

[4] Ross OA, Soto-Ortolaza AI, Heckman MG, Aasly JO, Abahuni N, Annesi G, et al (2011) Association of LRRK2 exonic variants with susceptibility to Parkinson's disease: a case-control study. Lancet Neurol 10: 898–908. 10.1016/S1474-4422(11)70175-2 21885347 PMC3208320

[5] Belin AC, Westerlund M (2008) Parkinson's disease: a genetic perspective. The FEBS journal 275: 1377–1383. 10.1111/j.1742-4658.2008.06301.x 18279377

[6] Marin I, van Egmond WN, van Haastert PJ (2008) The Roco protein family: a functional perspective. FASEB journal: official publication of the Federation of American Societies for Experimental Biology 22: 3103–3110. 10.1096/fj.08-111310 18523161

[7] Mata IF, Wedemeyer WJ, Farrer MJ, Taylor JP, Gallo KA (2006) LRRK2 in Parkinson's disease: protein domains and functional insights. Trends in neurosciences 29: 286–293. 16616379 10.1016/j.tins.2006.03.006

[8] Cookson MR (2010) The role of leucine-rich repeat kinase 2 (LRRK2) in Parkinson's disease. Nat Rev Neurosci 11: 791–797. 10.1038/nrn2935 21088684 PMC4662256

[9] Tsika E, Moore DJ (2012) Mechanisms of LRRK2-Mediated Neurodegeneration. Current neurology and neuroscience reports 12: 251–260. 10.1007/s11910-012-0265-8 22441981

[10] Kumar A, Cookson MR (2011) Role of LRRK2 kinase dysfunction in Parkinson disease. Expert reviews in molecular medicine 13: e20 10.1017/S146239941100192X 21676337 PMC4672634

[11] Kett LR, Dauer WT (2012) Leucine-rich repeat kinase 2 for beginners: six key questions. Cold Spring Harbor perspectives in medicine 2: a009407 10.1101/cshperspect.a009407 22393539 PMC3282500

[12] Yue Z, Lachenmayer ML (2011) Genetic LRRK2 models of Parkinson's disease: Dissecting the pathogenic pathway and exploring clinical applications. Movement disorders: official journal of the Movement Disorder Society 26: 1386–1397. 10.1002/mds.23737 21538530 PMC3150637

[13] Dachsel JC, Behrouz B, Yue M, Beevers JE, Melrose HL, Farrer MJ (2010) A comparative study of Lrrk2 function in primary neuronal cultures. Parkinsonism & related disorders 16: 650–655. 20850369 10.1016/j.parkreldis.2010.08.018 PMC3159957

[14] MacLeod D, Dowman J, Hammond R, Leete T, Inoue K, Abeliovich A (2006) The familial Parkinsonism gene LRRK2 regulates neurite process morphology. Neuron 52: 587–593. 17114044 10.1016/j.neuron.2006.10.008

[15] Plowey ED, Cherra SJ 3rd, Liu YJ, Chu CT (2008) Role of autophagy in G2019S-LRRK2-associated neurite shortening in differentiated SH-SY5Y cells. Journal of neurochemistry 105: 1048–1056. 10.1111/j.1471-4159.2008.05217.x 18182054 PMC2361385

[16] Winner B, Melrose HL, Zhao C, Hinkle KM, Yue M, Kent C, et al (2011) Adult neurogenesis and neurite outgrowth are impaired in LRRK2 G2019S mice. Neurobiology of disease 41: 706–716. 10.1016/j.nbd.2010.12.008 21168496 PMC3059106

[17] Ramonet D, Daher JP, Lin BM, Stafa K, Kim J, Banerjee R, et al (2011) Dopaminergic neuronal loss, reduced neurite complexity and autophagic abnormalities in transgenic mice expressing G2019S mutant LRRK2. PloS one 6: e18568 10.1371/journal.pone.0018568 21494637 PMC3071839

[18] Sanchez-Danes A, Richaud-Patin Y, Carballo-Carbajal I, Jimenez-Delgado S, Caig C, Mora S, et al (2012) Disease-specific phenotypes in dopamine neurons from human iPS-based models of genetic and sporadic Parkinson's disease. EMBO molecular medicine 4: 380–395. 10.1002/emmm.201200215 22407749 PMC3403296

[19] Parisiadou L, Cai H (2010) LRRK2 function on actin and microtubule dynamics in Parkinson disease. Communicative & integrative biology 3: 396–400. 21057624 10.4161/cib.3.5.12286 PMC2974064

[20] Parisiadou L, Xie C, Cho HJ, Lin X, Gu XL, Long CX, et al (2009) Phosphorylation of ezrin/radixin/moesin proteins by LRRK2 promotes the rearrangement of actin cytoskeleton in neuronal morphogenesis. The Journal of neuroscience: the official journal of the Society for Neuroscience 29: 13971–13980. 10.1523/JNEUROSCI.3799-09.2009 19890007 PMC2807632

[21] Chan D, Citro A, Cordy JM, Shen GC, Wolozin B (2011) Rac1 protein rescues neurite retraction caused by G2019S leucine-rich repeat kinase 2 (LRRK2). The Journal of biological chemistry 286: 16140–16149. 10.1074/jbc.M111.234005 21454543 PMC3091223

[22] Haebig K, Gloeckner CJ, Miralles MG, Gillardon F, Schulte C, Riess O, et al (2010) ARHGEF7 (Beta-PIX) acts as guanine nucleotide exchange factor for leucine-rich repeat kinase 2. PloS one 5: e13762 10.1371/journal.pone.0013762 21048939 PMC2966438

[23] Meixner A, Boldt K, Van Troys M, Askenazi M, Gloeckner CJ, Bauer M, et al (2011) A QUICK screen for Lrrk2 interaction partners—leucine-rich repeat kinase 2 is involved in actin cytoskeleton dynamics. Molecular & cellular proteomics: MCP 10: M110 001172. 10.1074/mcp.M110.001172 PMC3013447 20876399

[24] Deng X, Dzamko N, Prescott A, Davies P, Liu Q, Yang Q, et al (2011) Characterization of a selective inhibitor of the Parkinson's disease kinase LRRK2. Nature chemical biology 7: 203–205. 10.1038/nchembio.538 21378983 PMC3287420

[25] Sturchler-Pierrat C, Abramowski D, Duke M, Wiederhold KH, Mistl C, Rothacher S, et al (1997) Two amyloid precursor protein transgenic mouse models with Alzheimer disease-like pathology. Proc Natl Acad Sci U S A 94: 13287–13292. 9371838 10.1073/pnas.94.24.13287 PMC24301

[26] Klein CL, Rovelli G, Springer W, Schall C, Gasser T, Kahle PJ (2009) Homo- and heterodimerization of ROCO kinases: LRRK2 kinase inhibition by the LRRK2 ROCO fragment. J Neurochem 111: 703–715. 10.1111/j.1471-4159.2009.06358.x 19712061

[27] MacLeod DA, Rhinn H, Kuwahara T, Zolin A, Di Paolo G, McCabe BD, et al (2013) RAB7L1 interacts with LRRK2 to modify intraneuronal protein sorting and Parkinson's disease risk. Neuron 77: 425–439. 10.1016/j.neuron.2012.11.033 23395371 PMC3646583

[28] Sepulveda B, Mesias R, Li X, Yue Z, Benson DL (2013) Short- and long-term effects of LRRK2 on axon and dendrite growth. PLoS One 8: e61986 10.1371/journal.pone.0061986 23646112 PMC3640004

[29] Luerman GC, Nguyen C, Samaroo H, Loos P, Xi H, Hurtado-Lorenzo A, et al (2014) Phosphoproteomic evaluation of pharmacological inhibition of leucine-rich repeat kinase 2 reveals significant off-target effects of LRRK-2-IN-1. J Neurochem 128: 561–576. 10.1111/jnc.12483 24117733

[30] Biskup S, Moore DJ, Celsi F, Higashi S, West AB, Andrabi SA, et al (2006) Localization of LRRK2 to membranous and vesicular structures in mammalian brain. Annals of neurology 60: 557–569. 17120249 10.1002/ana.21019

[31] Biskup S, Moore DJ, Rea A, Lorenz-Deperieux B, Coombes CE, Dawson VL, et al (2007) Dynamic and redundant regulation of LRRK2 and LRRK1 expression. BMC neuroscience 8: 102 18045479 10.1186/1471-2202-8-102 PMC2233633

[32] Galter D, Westerlund M, Carmine A, Lindqvist E, Sydow O, Olson L (2006) LRRK2 expression linked to dopamine-innervated areas. Ann Neurol 59: 714–719. 16532471 10.1002/ana.20808

[33] Westerlund M, Belin AC, Anvret A, Bickford P, Olson L, Galter D (2008) Developmental regulation of leucine-rich repeat kinase 1 and 2 expression in the brain and other rodent and human organs: Implications for Parkinson's disease. Neuroscience 152: 429–436. 10.1016/j.neuroscience.2007.10.062 18272292

[34] Lin X, Parisiadou L, Gu XL, Wang L, Shim H, Sun L, et al (2009) Leucine-rich repeat kinase 2 regulates the progression of neuropathology induced by Parkinson's-disease-related mutant alpha-synuclein. Neuron 64: 807–827. 10.1016/j.neuron.2009.11.006 20064389 PMC2807409

[35] Melrose HL, Dachsel JC, Behrouz B, Lincoln SJ, Yue M, Hinkle KM, et al (2010) Impaired dopaminergic neurotransmission and microtubule-associated protein tau alterations in human LRRK2 transgenic mice. Neurobiology of disease 40: 503–517. 10.1016/j.nbd.2010.07.010 20659558 PMC2955774

[36] Xu Q, Shenoy S, Li C (2012) Mouse models for LRRK2 Parkinson's disease. Parkinsonism & related disorders 18 Suppl 1: S186–189. 22166430 10.1016/S1353-8020(11)70058-X

[37] Auburger G, Klinkenberg M, Drost J, Marcus K, Morales-Gordo B, Kunz WS, et al (2012) Primary skin fibroblasts as a model of Parkinson's disease. Mol Neurobiol 46: 20–27. 10.1007/s12035-012-8245-1 22350618 PMC3443476

[38] Vorotnikov AV (2011) Chemotaxis: movement, direction, control. Biochemistry Biokhimiia 76: 1528–1555. 10.1134/S0006297911130104 22339602

[39] Takeda M, Tanaka M, Kudo T, Nakamura Y, Tada K, Nishimura T (1990) Changes in adhesion efficiency and vimentin distribution of fibroblasts from familial Alzheimer's disease patients. Acta Neurol Scand 82: 238–244. 2270753 10.1111/j.1600-0404.1990.tb01613.x

[40] Stacey NC, Ma MH, Duley JA, Connolly GP (2000) Abnormalities in cellular adhesion of neuroblastoma and fibroblast models of Lesch Nyhan syndrome. Neuroscience 98: 397–401. 10854773 10.1016/s0306-4522(00)00149-4

