# Supplementary figures and images for: No Dopamine Cell Loss or Changes in Cytoskeleton Function in Transgenic Mice Expressing Physiological Levels of Wild Type or G2019S Mutant LRRK2 and in Human Fibroblasts
Source: PLoS One. 2015 Apr 1;10(4):e0118947. doi: 10.1371/journal.pone.0118947 (PMC4382199; doi:10.1371/journal.pone.0118947)

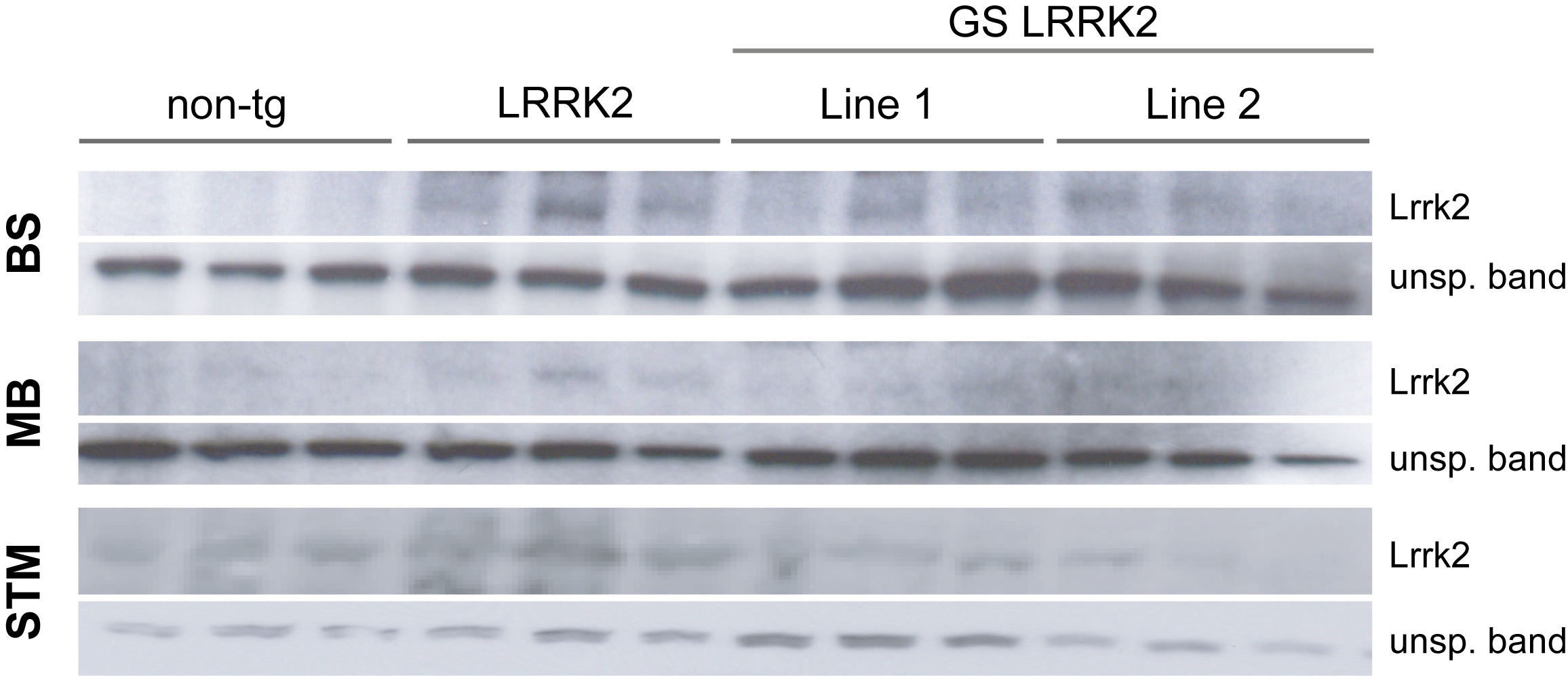

Supplement: S1 Fig — Western blot analysis of LRRK2 protein showed expression levels of LRRK2 in brainstem (BS), midbrain (MB), but not in striatum (STM) of 10-month-old animals with the human-specific LRRK2 antibody Novus 267. n = 3 animals per genotype. (TIF) [file pone.0118947.s001.tif]

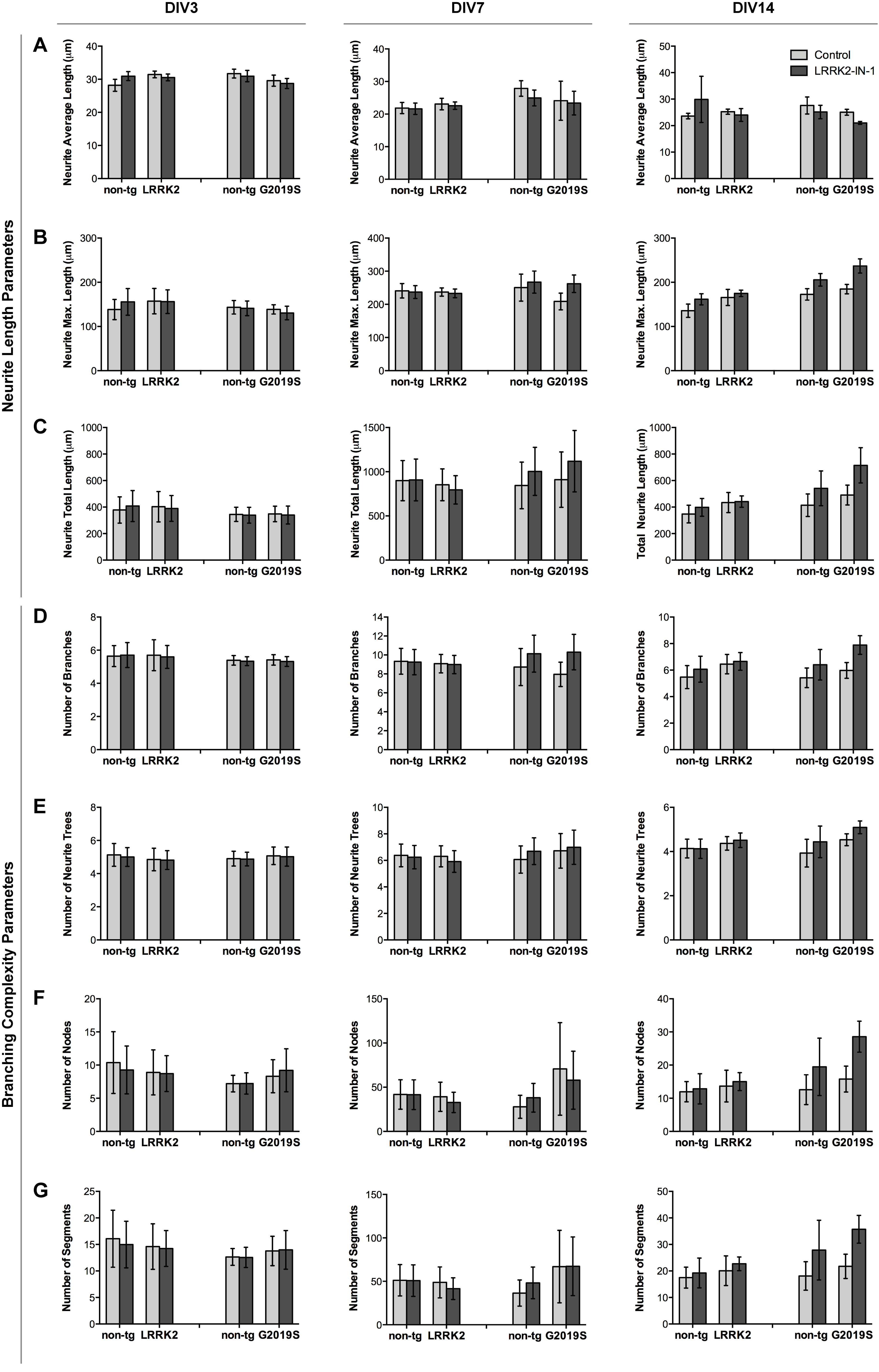

Supplement: S2 Fig — A-G: Neurite parameters analyzed in neuronal cultures from LRRK2, GS-LRRK2 (line 2), and their respective non-tg littermate neurons which were treated with vehicle-control or LRRK2-IN-1 (0.1 μM) for three (DIV3), seven (DIV7), or fourteen days (DIV14). Data represent mean ± SEM; Two-way ANOVA (* p<0.05; ** p<0.01); Number of neurons analyzed for cultures obtained from LRRK2 transgenic mice: non-tg = 1339, non-tg + LRRK2-IN-1 = 1609; wild type LRRK2 = 1697, wild type LRRK2 + LRRK2-IN-1 = 1542, n = 4 independent experiments; Number of neurons analyzed for cultures obtained from GS-LRRK2 transgenic mice: non-tg = 1268; non-tg + LRRK2-IN-1 = 1522; GS-LRRK2 = 1526; GS-LRRK2 + LRRK2-IN-1 = 1844, n = 4 independent experiments. (TIF) [file pone.0118947.s002.tif]

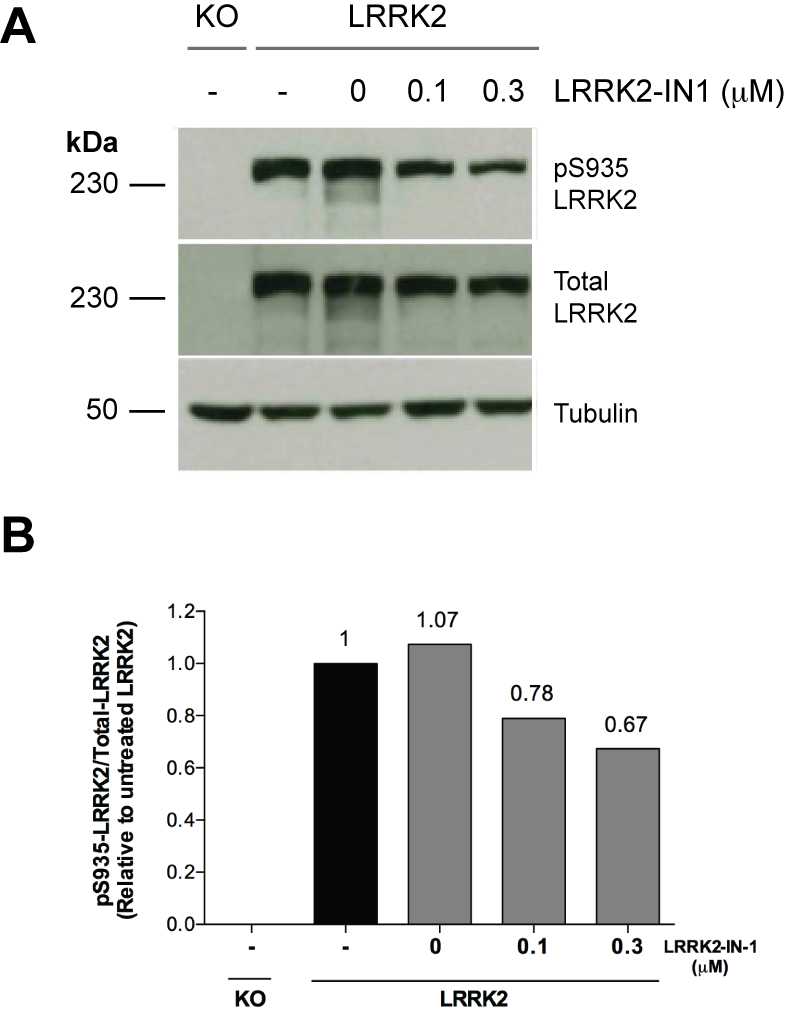

Supplement: S3 Fig — A: Western blot analysis showing reduction of endogenous LRRK2 phosphorylation at residue S935 in mouse fibroblasts after incubation with LRRK2-IN1 inhibitor at different concentrations (0, 0.1, and 0.3 μM) for 20 min. Inhibition of LRRK2 kinase activity can be observed at 0.1 μM of LRRK2-IN1 by reducing LRRK2 phosphorylation at residue S935 (phosphospecific LRRK2 antibody against residue pS935). B: Densitometry quantification revealed approximately 22% reduction of pS935 LRRK2 protein after incubation with 0.1μM of LRRK2-IN1. (TIF) [file pone.0118947.s003.tif]

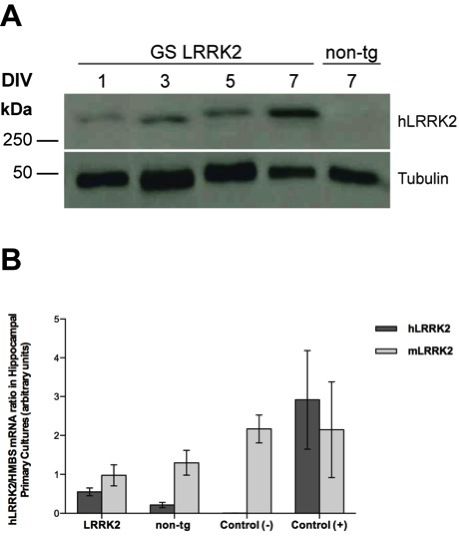

Supplement: S4 Fig — A: Western blot analysis of human LRRK2 protein expression in GS-LRRK2 primary hippocampal cultures at DIV1, 3, 5 and 7 derived from GS-LRRK2 transgenic newborn pups. Human LRRK2 protein is only expressed in GS-LRRK2 primary hippocampal neurons but not in their non-tg littermate controls (human-specific anti-LRRK2 antibody MJFF5). B: RT-PCR semi-quantification of human LRRK2 mRNA expression in LRRK2 primary hippocampal cultures at DIV7 derived from LRRK2 transgenic newborn pups and their respective non-tg littermates. Whole brain lysate from non-transgenic and LRRK2 transgenic mice were used as negative and positive control, respectively. Data represents mean ± SEM. Number of newborn pups: non-tg = 5, LRRK2 = 7; Number of control samples: negative = 2, positive = 2; n = 2 independent experiments. (JPG) [file pone.0118947.s004.jpg]

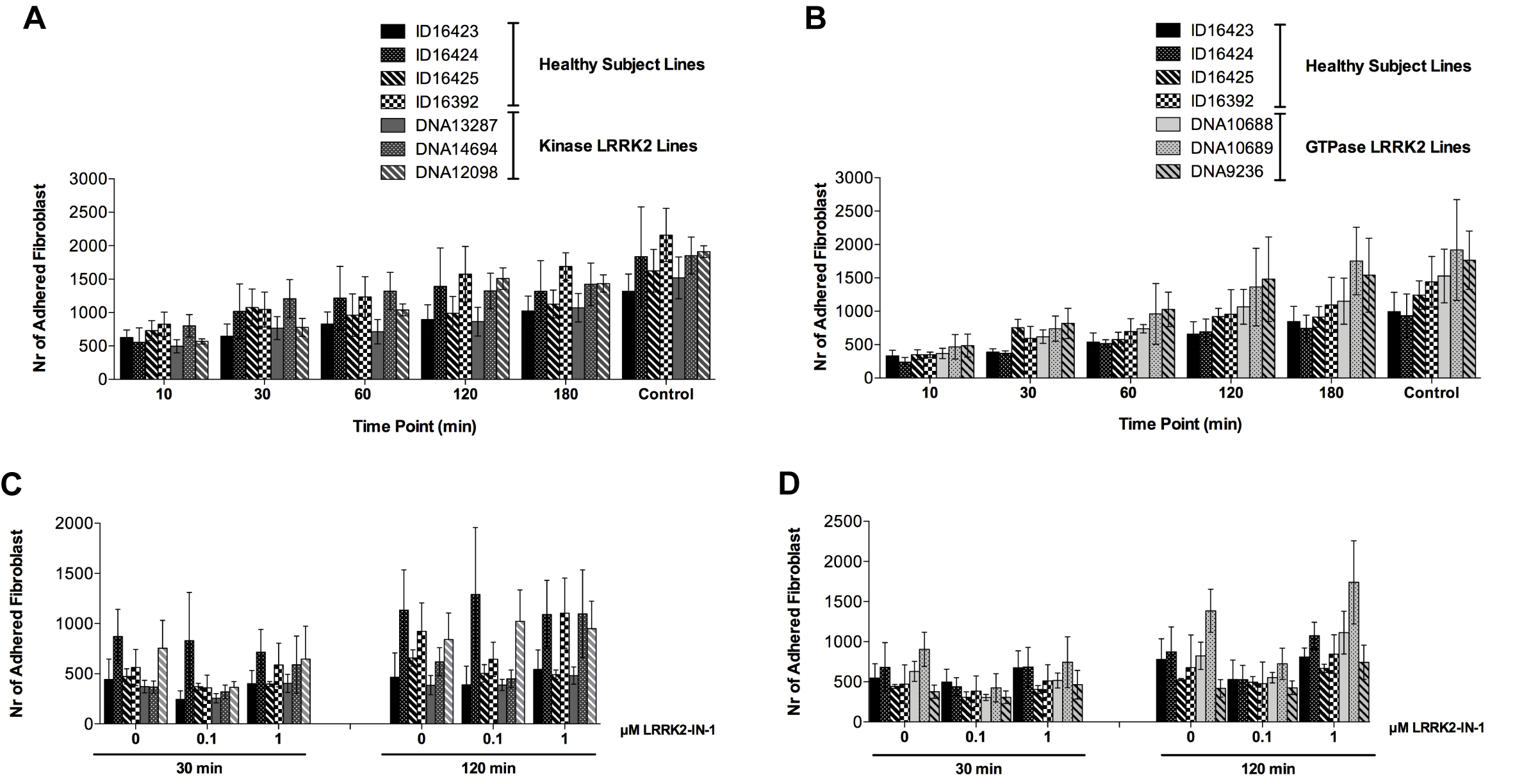

Supplement: S5 Fig — A-B: Percentage of adhered fibroblasts with wild type LRRK2 (healthy subject lines) and LRRK2 mutations in the kinase (A) and ROC (B) domain at different time points. C-D: Percentage of adhered fibroblasts with wild type LRRK2 (healthy subject lines) and LRRK2 mutations in the kinase (C) and ROC (D) domain after treatment with vehicle control (0), 0.1μM or 1μM LRRK2-IN-1 for 30 and 120 minutes. Data represent mean ± SEM; n = 4 independent experiments (A, B) and n = 3 independent experiments (C, D). Healthy-Subjects (wild type LRRK2) = 4 lines; G2019S LRRK2 patients (GS) = 3 lines; N1437S LRRK2 patient (NS) = 2 lines; R1441C LRRK2 patients (RC) = 1 line. (Two-way ANOVA with Repeated Measures). (TIF) [file pone.0118947.s005.tif]
