# Supplementary material for: No Dopamine Cell Loss or Changes in Cytoskeleton Function in Transgenic Mice Expressing Physiological Levels of Wild Type or G2019S Mutant LRRK2 and in Human Fibroblasts
Source: PLoS One. 2015 Apr 1;10(4):e0118947. doi: 10.1371/journal.pone.0118947 (PMC4382199; doi:10.1371/journal.pone.0118947)
Supplement: S1 File — Table A, Two-way ANOVA statistical results from primary hippocampal neurons at DIV3. Table B, Two-way ANOVA statistical results from primary hippocampal neurons at DIV7. Table C, Two-way ANOVA statistical results from primary hippocampal neurons at DIV14. Table D, Two-way ANOVA statistical results from cellular adhesion assay timeline. Table E, Two-way ANOVA statistical results from cellular adhesion assay LRRK2-IN-1 treatment. (DOCX) [file pone.0118947.s006.docx]

**S6. Statistic Table Set**

| **Primary Hippocampal Neurons – DIV3** | | |
| --- | --- | --- |
| **Two-way ANOVA** | **Ordinary** |  |
| **Alpha** | **0.05** |  |
| **DIV3-Nr of Branches-WT LRRK2** | | |
|  | **F (DFn, DFd)** | **P value** |
| Interaction | F (1, 16) = 0.01165 | P = 0.9154 |
| Genotype | F (1, 16) = 0.001560 | P = 0.9690 |
| Treatment | F (1, 16) = 0.0006932 | P = 0.9793 |
| **DIV3-Nr of Branches-G2019S LRRK2** | | |
|  | **F (DFn, DFd)** | **P value** |
| Interaction | F (1, 8) = 0.005685 | P = 0.9418 |
| Genotype | F (1, 8) = 0.0002167 | P = 0.9886 |
| Treatment | F (1, 8) = 0.06273 | P = 0.8085 |
| **DIV3-Neurite Max Length-WT LRRK2** | | |
|  | **F (DFn, DFd)** | **P value** |
| Interaction | F (1, 16) = 0.1150 | P = 0.7389 |
| Genotype | F (1, 16) = 0.1294 | P = 0.7238 |
| Treatment | F (1, 16) = 0.08650 | P = 0.7725 |
| **DIV3-Neurite Max Length-G2019S LRRK2** | | |
|  | **F (DFn, DFd)** | **P value** |
| Interaction | F (1, 8) = 0.04188 | P = 0.8430 |
| Genotype | F (1, 8) = 0.2782 | P = 0.6122 |
| Treatment | F (1, 8) = 0.1283 | P = 0.7294 |
| **DIV3-Neurite Total Length-WT LRRK2** | | |
|  | **F (DFn, DFd)** | **P value** |
| Interaction | F (1, 16) = 0.04034 | P = 0.8433 |
| Genotype | F (1, 16) = 0.0009677 | P = 0.9756 |
| Treatment | F (1, 16) = 0.006152 | P = 0.9385 |
| **DIV3-Neurite Total Length-G2019S LRRK2** | | |
|  | **F (DFn, DFd)** | **P value** |
| Interaction | F (1, 8) = 0.0002003 | P = 0.9891 |
| Genotype | F (1, 8) = 0.001275 | P = 0.9724 |
| Treatment | F (1, 8) = 0.01438 | P = 0.9075 |
| **DIV3-Nr of Trees-WT LRRK2** | | |
|  | **F (DFn, DFd)** | **P value** |
| Interaction | F (1, 16) = 0.005141 | P = 0.9437 |
| Genotype | F (1, 16) = 0.1402 | P = 0.7130 |
| Treatment | F (1, 16) = 0.01505 | P = 0.9039 |
| **DIV3-Nr of Trees-G2019S LRRK2** | | |
|  | **F (DFn, DFd)** | **P value** |
| Interaction | F (1, 8) = 0.0002009 | P = 0.9890 |
| Genotype | F (1, 8) = 0.1031 | P = 0.7564 |
| Treatment | F (1, 8) = 0.006165 | P = 0.9393 |
| **DIV3-Nr of Nodes-WT LRRK2** | | |
|  | **F (DFn, DFd)** | **P value** |
| Interaction | F (1, 16) = 0.01621 | P = 0.9003 |
| Genotype | F (1, 16) = 0.07738 | P = 0.7844 |
| Treatment | F (1, 16) = 0.03155 | P = 0.8613 |
| **DIV3-Nr of Nodes-G2019S LRRK2** | | |
|  | **F (DFn, DFd)** | **P value** |
| Interaction | F (1, 8) = 0.03643 | P = 0.8534 |
| Genotype | F (1, 8) = 0.4568 | P = 0.5182 |
| Treatment | F (1, 8) = 0.04093 | P = 0.8447 |
| **DIV3-Avg Length-WT LRRK2** | | |
|  | **F (DFn, DFd)** | **P value** |
| Interaction | F (1, 16) = 1.911 | P = 0.1859 |
| Genotype | F (1, 16) = 1.170 | P = 0.2955 |
| Treatment | F (1, 16) = 0.4956 | P = 0.4916 |
| **DIV3-Avg Length-G2019S LRRK2** | | |
|  | **F (DFn, DFd)** | **P value** |
| Interaction | F (1, 8) = 0.0006936 | P = 0.9796 |
| Genotype | F (1, 8) = 1.937 | P = 0.2015 |
| Treatment | F (1, 8) = 0.2618 | P = 0.6227 |
| **DIV3-Nr of Segments-WT LRRK2** | | |
|  | **F (DFn, DFd)** | **P value** |
| Interaction | F (1, 16) = 0.006866 | P = 0.9350 |
| Genotype | F (1, 16) = 0.06360 | P = 0.8041 |
| Treatment | F (1, 16) = 0.02776 | P = 0.8698 |
| **DIV3-Nr of Segments-G2019S LRRK2** | | |
|  | **F (DFn, DFd)** | **P value** |
| Interaction | F (1, 8) = 0.003319 | P = 0.9555 |
| Genotype | F (1, 8) = 0.2389 | P = 0.6381 |
| Treatment | F (1, 8) = 0.0003947 | P = 0.9846 |

**Table A. Two-way ANOVA statistical results from primary hippocampal neurons at DIV3.**

| **Primary Hippocampal Neurons – DIV7** | | |
| --- | --- | --- |
| **Two-way ANOVA** | **Ordinary** |  |
| **Alpha** | **0.05** |  |
| **DIV7-Nr of Branches-WT LRRK2** | | |
|  | **F (DFn, DFd)** | **P value** |
| Interaction | F (1, 16) = 1.122e-005 | P = 0.9974 |
| Genotype | F (1, 16) = 0.04629 | P = 0.8324 |
| Treatment | F (1, 16) = 0.005729 | P = 0.9406 |
| **DIV7-Nr of Branches- G2019S LRRK2** | | |
|  | **F (DFn, DFd)** | **P value** |
| Interaction | F (1, 12) = 0.06892 | P = 0.7974 |
| Genotype | F (1, 12) = 0.02852 | P = 0.8687 |
| Treatment | F (1, 12) = 1.107 | P = 0.3134 |
| **DIV7-Neurite Max Length-WT LRRK2** | | |
|  | **F (DFn, DFd)** | **P value** |
| Interaction | F (1, 16) = 6.911e-005 | P = 0.9935 |
| Genotype | F (1, 16) = 0.05185 | P = 0.8228 |
| Treatment | F (1, 16) = 0.05451 | P = 0.8184 |
| **DIV7-Neurite Max Length-G2019S LRRK2** | | |
|  | **F (DFn, DFd)** | **P value** |
| Interaction | F (1, 12) = 0.3289 | P = 0.5769 |
| Genotype | F (1, 12) = 0.5307 | P = 0.4803 |
| Treatment | F (1, 12) = 1.200 | P = 0.2948 |
| **DIV7-Nr of Trees-WT LRRK2** | | |
|  | **F (DFn, DFd)** | **P value** |
| Interaction | F (1, 16) = 0.02392 | P = 0.8790 |
| Genotype | F (1, 16) = 0.05785 | P = 0.8130 |
| Treatment | F (1, 16) = 0.1003 | P = 0.7556 |
| **DIV7-Nr of Trees-G2019S LRRK2** | | |
|  | **F (DFn, DFd)** | **P value** |
| Interaction | F (1, 12) = 0.02380 | P = 0.8800 |
| Genotype | F (1, 12) = 0.1676 | P = 0.6894 |
| Treatment | F (1, 12) = 0.1458 | P = 0.7093 |
| **DIV7-Nr of Nodes-WT LRRK2** | | |
|  | **F (DFn, DFd)** | **P value** |
| Interaction | F (1, 16) = 0.03899 | P = 0.8460 |
| Genotype | F (1, 16) = 0.1308 | P = 0.7223 |
| Treatment | F (1, 16) = 0.04580 | P = 0.8332 |
| **DIV7-Nr of Nodes-G2019S LRRK2** | | |
|  | **F (DFn, DFd)** | **P value** |
| Interaction | F (1, 12) = 0.1249 | P = 0.7299 |
| Genotype | F (1, 12) = 0.9243 | P = 0.3553 |
| Treatment | F (1, 12) = 0.001579 | P = 0.9690 |
| **DIV7-Avg Length-WT LRRK2** | | |
|  | **F (DFn, DFd)** | **P value** |
| Interaction | F (1, 16) = 0.006454 | P = 0.9370 |
| Genotype | F (1, 16) = 0.4570 | P = 0.5087 |
| Treatment | F (1, 16) = 0.05213 | P = 0.8223 |
| **DIV7-Avg Length-G2019S LRRK2** | | |
|  | **F (DFn, DFd)** | **P value** |
| Interaction | F (1, 12) = 0.08047 | P = 0.7815 |
| Genotype | F (1, 12) = 0.4668 | P = 0.5074 |
| Treatment | F (1, 12) = 0.2196 | P = 0.6477 |
| **DIV7-Nr of Segments-WT LRRK2** | | |
|  | **F (DFn, DFd)** | **P value** |
| Interaction | F (1, 16) = 0.03955 | P = 0.8449 |
| Genotype | F (1, 16) = 0.1202 | P = 0.7333 |
| Treatment | F (1, 16) = 0.05166 | P = 0.8231 |
| **DIV7-Nr of Segments-G2019S LRRK2** | | |
|  | **F (DFn, DFd)** | **P value** |
| Interaction | F (1, 12) = 0.03811 | P = 0.8485 |
| Genotype | F (1, 12) = 0.7148 | P = 0.4144 |
| Treatment | F (1, 12) = 0.04272 | P = 0.8397 |
| **DIV7-Neurite Total Length-WT LRRK2** | | |
|  | **F (DFn, DFd)** | **P value** |
| Interaction | F (1, 16) = 0.02669 | P = 0.8723 |
| Genotype | F (1, 16) = 0.1554 | P = 0.6986 |
| Treatment | F (1, 16) = 0.01532 | P = 0.9030 |
| **DIV7-Neurite Total Length-G2019S LRRK2** | | |
|  | **F (DFn, DFd)** | **P value** |
| Interaction | F (1, 12) = 0.006702 | P = 0.9361 |
| Genotype | F (1, 12) = 0.08987 | P = 0.7695 |
| Treatment | F (1, 12) = 0.3751 | P = 0.5517 |

**Table B. Two-way ANOVA statistical results from primary hippocampal neurons at DIV7**

| **Primary Hippocampal Neurons – DIV14** | | |
| --- | --- | --- |
| **Two-way ANOVA** | **Ordinary** |  |
| **Alpha** | **0.05** |  |
| **Nr of Branches-WT LRRK2** | |  |
|  | **F (DFn, DFd)** | **P value** |
| Interaction | F (1, 8) = 0.05553 | P = 0.8196 |
| Genotype | F (1, 8) = 0.9155 | P = 0.3667 |
| Treatment | F (1, 8) = 0.2422 | P = 0.6358 |
| **Nr of Branches-G2019S LRRK2** | | |
|  | **F (DFn, DFd)** | **P value** |
| Interaction | F (1, 8) = 0.3186 | P = 0.5879 |
| Genotype | F (1, 8) = 1.529 | P = 0.2514 |
| Treatment | F (1, 8) = 3.090 | P = 0.1168 |
| **Neurite Max Length-WT LRRK2** | | |
|  | **F (DFn, DFd)** | **P value** |
| Interaction | F (1, 8) = 0.3549 | P = 0.5678 |
| Genotype | F (1, 8) = 2.413 | P = 0.1589 |
| Treatment | F (1, 8) = 1.603 | P = 0.2411 |
| **Neurite Max Length-G2019S LRRK2** | | |
|  | **F (DFn, DFd)** | P value |
| Interaction | F (1, 8) = 0.5174 | P = 0.4924 |
| Genotype | F (1, 8) = 2.563 | P = 0.1480 |
| Treatment | F (1, 8) = 9.898 | P = 0.0137 |
| **Nr of Trees-WT LRRK2** | | |
|  | **F (DFn, DFd)** | **P value** |
| Interaction | F (1, 8) = 0.04095 | P = 0.8447 |
| Genotype | F (1, 8) = 0.6727 | P = 0.4359 |
| Treatment | F (1, 8) = 0.03098 | P = 0.8647 |
| **Nr of Trees-G2019S LRRK2** | | |
|  | **F (DFn, DFd)** | **P value** |
| Interaction | F (1, 8) = 0.002237 | P = 0.9634 |
| Genotype | F (1, 8) = 1.504 | P = 0.2549 |
| Treatment | F (1, 8) = 1.089 | P = 0.3272 |
| **Nr of Nodes-WT LRRK2** | | |
|  | **F (DFn, DFd)** | **P value** |
| Interaction | F (1, 8) = 0.003676 | P = 0.9531 |
| Genotype | F (1, 8) = 0.2488 | P = 0.6313 |
| Treatment | F (1, 8) = 0.08306 | P = 0.7805 |
| **Nr of Nodes-G2019S LRRK2** | | |
|  | **F (DFn, DFd)** | **P value** |
| Interaction | F (1, 8) = 0.2630 | P = 0.6219 |
| Genotype | F (1, 8) = 1.146 | P = 0.3157 |
| Treatment | F (1, 8) = 2.928 | P = 0.1254 |
| **Avg Length-WT LRRK2** | | |
|  | **F (DFn, DFd)** | **P value** |
| Interaction | F (1, 8) = 0.6722 | P = 0.4360 |
| Genotype | F (1, 8) = 0.2121 | P = 0.6574 |
| Treatment | F (1, 8) = 0.3065 | P = 0.5950 |
| **Avg Length-G2019S LRRK2** | | |
|  | **F (DFn, DFd)** | **P value** |
| Interaction | F (1, 8) = 0.1309 | P = 0.7269 |
| Genotype | F (1, 8) = 2.441 | P = 0.1568 |
| Treatment | F (1, 8) = 2.322 | P = 0.1661 |
| **Nr of Segments-WT LRRK2** | | |
|  | **F (DFn, DFd)** | **P value** |
| Interaction | F (1, 8) = 0.008774 | P = 0.9277 |
| Genotype | F (1, 8) = 0.4296 | P = 0.5306 |
| Treatment | F (1, 8) = 0.2219 | P = 0.6502 |
| **Nr of Segments-G2019S LRRK2** | | |
|  | **F (DFn, DFd)** | **P value** |
| Interaction | F (1, 8) = 0.08754 | P = 0.7749 |
| Genotype | F (1, 8) = 0.6515 | P = 0.4429 |
| Treatment | F (1, 8) = 2.767 | P = 0.1348 |
| **Neurite Total Length-WT LRRK2** | | |
|  | **F (DFn, DFd)** | **P value** |
| Interaction | F (1, 8) = 0.1106 | P = 0.7480 |
| Genotype | F (1, 8) = 1.038 | P = 0.3382 |
| Treatment | F (1, 8) = 0.2091 | P = 0.6596 |
| **Neurite Total Length-G2019S LRRK2** | | |
|  | **F (DFn, DFd)** | **P value** |
| Interaction | F (1, 8) = 0.1970 | P = 0.6690 |
| Genotype | F (1, 8) = 1.313 | P = 0.2850 |
| Treatment | F (1, 8) = 2.600 | P = 0.1455 |

**Table C. Two-way ANOVA statistical results from primary hippocampal neurons at DIV14.**

| **Cellular Adhesion Assay Timeline** | | | | |
| --- | --- | --- | --- | --- |
| **Two-way RM ANOVA** | | **Matching: Stacked** | |  |
| **Alpha** | **0.05** | |  | |
| **GTPase Timeline** | | | | |
|  | **F (DFn, DFd)** | | **P value** | |
| **Interaction** | F (10, 25) = 0.7501 | | P = 0.6727 | |
| **Time** | F (5, 25) = 11.71 | | P < 0.0001 | |
| **LRRK2 Genotype** | F (2, 5) = 0.7465 | | P = 0.5204 | |
| **Subjects (matching)** | F (5, 25) = 8.631 | | P < 0.0001 | |
| **Kinase Timeline** | | | | |
|  | **F (DFn, DFd)** | | **P value** | |
| **Interaction** | F (5, 30) = 0.1255 | | P = 0.9855 | |
| **Time** | F (5, 30) = 52.85 | | P < 0.0001 | |
| **LRRK2 Genotype** | F (1, 6) = 2.345e-005 | | P = 0.9963 | |
| **Subjects (matching)** | F (6, 30) = 43.26 | | P < 0.0001 | |

**Table D. Two-way ANOVA statistical results from cellular adhesion assay timeline.**

| **Cellular Adhesion Assay LRRK2-IN-1 Treatment** | | | | |
| --- | --- | --- | --- | --- |
| **Two-way ANOVA** | **Ordinary** |  |  |  |
| **Alpha** | **0.05** |  |  |  |
|  | **GTPase-30 min** | | **Kinase-30 min** | |
|  | **F (DFn, DFd)** | **P value** | **F (DFn, DFd)** | **P value** |
| **Interaction** | F (4, 18) = 0.2453 | P = 0.9088 | F (2, 17) = 1.219 | P = 0.3201 |
| **Treatment** | F (2, 18) = 1.594 | P = 0.2304 | F (2, 17) = 2.663 | P = 0.0986 |
| **LRRK2 Genotype** | F (2, 18) = 1.317 | P = 0.2925 | F (1, 17) = 0.5234 | P = 0.4792 |
|  | **GTPase-120 min** | | **Kinase-120 min** | |
|  | **F (DFn, DFd)** | **P value** | **F (DFn, DFd)** | **P value** |
| **Interaction** | F (4, 18) = 0.5886 | P = 0.6751 | F (2, 18) = 0.3139 | P = 0.7345 |
| **Treatment** | F (2, 18) = 4.245 | P = 0.0309 | F (2, 18) = 2.296 | P = 0.1294 |
| **LRRK2 Genotype** | F (2, 18) = 5.612 | P = 0.0128 | F (1, 18) = 2.016 | P = 0.1728 |

**Table E. Two-way ANOVA statistical results from cellular adhesion assay LRRK2-IN-1 treatment.**
