# Supplementary material for: No Dopamine Cell Loss or Changes in Cytoskeleton Function in Transgenic Mice Expressing Physiological Levels of Wild Type or G2019S Mutant LRRK2 and in Human Fibroblasts
Source: PLoS One. 2015 Apr 1;10(4):e0118947. doi: 10.1371/journal.pone.0118947 (PMC4382199; doi:10.1371/journal.pone.0118947)
Supplement: S1 Protocol — B, LRRK2 kinase inhibition by LRRK2-IN1 in mouse fibroblasts. (DOCX) [file pone.0118947.s007.docx]

**S7. Protocol Word File**

**S7 Protocol A. LRRK2 protein quantification in primary hippocampal cultures.** Primary hippocampal cultures were prepared as described in Material and Methods from GS- LRRK2 transgenic newborn pups. At day in vitro (DIV) 1, 3, 5 and 7 neuronal cultures were homogenized in freshly prepared lysis buffer (1% Triton X-100, 1X complete protease inhibitor Cocktail [Roche], and 1X Phosphatase inhibitor cocktail [Roche] in PBS) and protein concentration was determined using the BCA protein Assay Kit (Pierce Thermo Scientific) following the manufacturer’s instructions. Samples were resolved using 8% acrylamide SDS-PAGE, transfer onto polyvinylidenfluorid (PDVF) membranes (Millipore) and incubated overnight at 4°C with primary antibody anti-LRRK2 MJFF5 (Clone c68-7) and anti-ß-tubulin III (Sigma-Aldrich). Horseradish peroxidase-conjugated secondary antibodies anti-mouse (Jackson Immunoresearch) or anti-rabbit (Cell Signaling) and chemiluminiscence HRP substrate (Immobilion Western HRP substrate) were used for detection.

**S7 Protocol B. LRRK2 kinase inhibition by LRRK2-IN1 in mouse fibroblasts.** Mouse fibroblasts 48.000 cells/well were incubated for 20 min with vehicle (DMSO), 0.1 or 0.3 µM of LRRK2-IN-1 in DMSO. Fibroblasts were homogenized in freshly prepared lysis buffer (1% Triton X-100 in PBS) and protein concentration was determined as described above. Samples were resolved using 8% acrylamide SDS-PAGE, transfer onto polyvinylidenfluorid (PDVF) membranes (Millipore) and incubated overnight at 4°C with primary antibody anti-LRRK2 MJFF2 (Clone c41-2), anti-LRRK2 S935 (Epitomics), and anti-ß-tubulin III (Sigma-Aldrich). Horseradish peroxidase-conjugated secondary antibodies anti-mouse (Jackson Immunoresearch) or anti-rabbit (Cell Signaling) and chemiluminiscence HRP substrate (Immobilion Western HRP substrate) were used for detection.
